# Evolution of a theory of mind

**DOI:** 10.1016/j.isci.2024.108862

**Published:** 2024-01-11

**Authors:** Tom Lenaerts, Marco Saponara, Jorge M. Pacheco, Francisco C. Santos

**Affiliations:** 1 Machine Learning Group, Département d’Informatique, Université Libre de Bruxelles, 1050 Brussels, Belgium; 2 Artificial Intelligence Lab, Vakgroep Computerwetenschappen, Vrije Universiteit Brussel, 1050 Brussels, Belgium; 3 Center for Human-Compatible AI, University of California, Berkeley, Berkeley, CA 94702, USA; 4 Centro de Biologia Molecular e Ambiental, Universidade do Minho, 4710 - 057 Braga, Portugal; 5 Departamento de Matemática e Aplicações, Universidade do Minho, 4710 - 057 Braga, Portugal; 6 ATP-group, P-2744-016 Porto Salvo, Portugal; 7 INESC-ID and Instituto Superior Técnico, Universidade de Lisboa, IST-Taguspark, 2744-016 Porto Salvo, Portugal

**Keywords:** Behavioral neuroscience, cognitive neuroscience, game playing

## Abstract

Even though the Theory of Mind in upper primates has been under investigation for decades, how it may evolve remains an open problem. We propose here an evolutionary game theoretical model where a finite population of individuals may use reasoning strategies to infer a response to the anticipated behavior of others within the context of a sequential dilemma, i.e., the Centipede Game. We show that strategies with bounded reasoning evolve and flourish under natural selection, provided they are allowed to make reasoning mistakes and a temptation for higher future gains is in place. We further show that non-deterministic reasoning co-evolves with an optimism bias that may lead to the selection of new equilibria, closely associated with average behavior observed in experimental data. This work reveals both a novel perspective on the evolution of bounded rationality and a co-evolutionary link between the evolution of Theory of Mind and the emergence of misbeliefs.

## Highlights

• Boundedly rational strategies evolve readily in the incremental centipede game
• Strategies with the theory of mind change the centipede game into coordination game
• Intermediate levels of reasoning co-evolve with optimistic beliefs about others
• The uncertainty about future gains is vital for the evolution of theory of mind

## Graphical abstract

**Figure:**
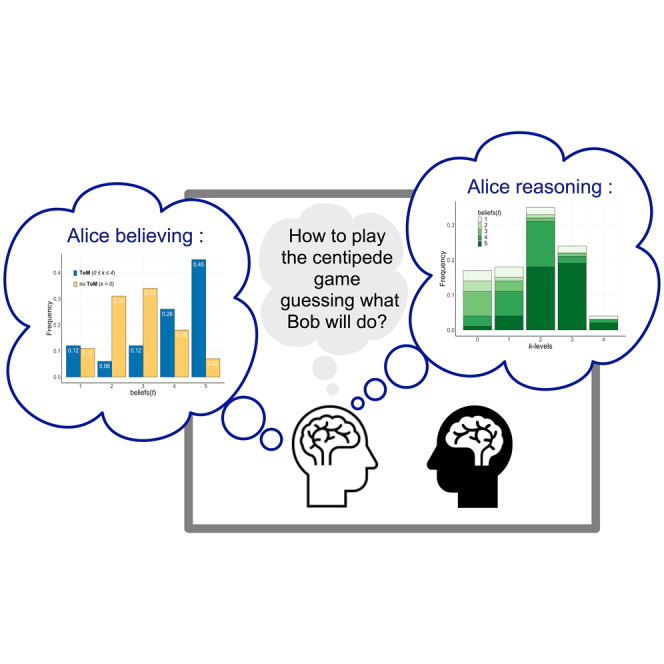

## Introduction

Social cognition is fundamental to human decision-making.^1^^,^^2^ It allows people to consider the beliefs, desires, and intentions of others when making choices within social interactions.^3^^,^^4^^,^^5^^,^^6^^,^^7^ The ability to see others as beings with mental states and reason about them is called Theory of Mind (ToM),^8^ which covers a wide spectrum of cognitive and affective capacities. Possessing ToM is considered to be beneficial since it allows one to anticipate (and potentially outsmart or align with) the behavior of others, selecting consequently actions that are most appropriate in a given social situation. Neurological disorders such as autism spectrum disorder have been linked to the impairment of ToM,^3^^,^^9^^,^^10^^,^^11^^,^^12^ where affected individuals have difficulties in assigning internal states to, or recognizing emotions expressed by, others.

Despite many experiments performed to assess the level of ToM in different species,^13^^,^^14^ things remain unclear regarding its evolutionary origins. The current consensus appears to be that ToM evolved as a consequence of the increasingly complex social interactions that early humans had to deal with^3^^,^^5^^,^^15^^,^^16^^,^^17^^,^^18^: Living in groups required humans to have the ability to discern who may be cooperative and who may defect, acquiring thus the capacity to identify honesty and deception and to act accordingly. Alternative theories continue to appear^19^ as there is no actual data to construct the real picture about ToM’s origin. In general, the relevance of ToM has been argued both from a competitive^5^ and cooperative^20^ perspective, with the latter being considered to have produced the more sophisticated ToM that is associated with human intelligence.^7^

Lacking historical data, potential evolutionary routes to ToM may be explored through evolutionary models^21^^,^^22^^,^^23^^,^^24^: Early work by Stahl examined via evolutionary game theory (EGT) methods for infinite populations whether smarter players, modeled as a hierarchy of best-response strategies, could outcompete the less smart ones.^21^ An extreme assumption of this model was that all players were perfectly informed about the distribution of smartness in the population (or at least about the distribution of smartness of players less smart than themselves). His work showed that smartness expressed as a higher ToM level does not lead to superior fitness and concluded that being right – i.e., taking the right action – is as good as being smart. Devaine et al.^23^ demonstrated, in a replicator model where individuals are endowed with a sophisticated meta-Bayesian mechanism to update beliefs and forecast the behavior of other players,^25^ that individuals with such higher-level Bayesian ToM benefit from competitive games as opposed to cooperative ones, where the former apparently contradict Stahl’s earlier results. Their observations strongly depend on a Bayes-optimality assumption, which, while aligning with the classic rationality assumptions in economic agents, is at odds with many observations on biases in human decision-making. More recently, Qi and Vul^24^ analyzed a similar idea, asking what type of environment is more conducive to the evolution of a Bayesian ToM agent, comparing it with several fixed-action agents as well as those using other types of inference or learning to determine a co-player’s attitude toward the focal player. They showed that uncertainty in the game environment leads to the dominance of such a Bayesian ToM strategy, stressing the importance of uncertainty about the outcomes in environments for the evolution of ToM as in Rusch et al.^2^

Our work differs from these lines of research in that we do not make strong assumptions about the knowledge of individuals nor about their predictive capacity concerning some hidden characteristic of co-players. We investigate to which extent, within the framework of evolutionary game theory in finite populations, individuals that have personal beliefs about the behavior of others and can reason (or not) about these beliefs with different levels of sophistication lead to the evolution of sophisticated reasoning capacities. In this quest, we further analyze what they believe in and how they act. While strategies have predefined beliefs and different levels of sophistication in their reasoning, they will use all the same reasoning process (RP) to arrive at their choices, as is specified in detail later in discussion. The present model does not consider other elements, such as belief updating or attitude prediction in repeated interactions, although its extension to include such features is feasible. At this stage, however, no comprehensive answers to the previously mentioned basic questions have been provided in the literature.

To answer the question above, one needs to rely on social decision processes that are relevant to the study of ToM,^2^ i.e., tasks with a level of ambiguity or uncertainty about the effects of the actions on the environment or the anticipated outcomes, on the one hand, and wherein interdependent thought processes are key, on the other hand. One of the many abstract tasks that fits these requirements and will serve thus as our workhorse here is the Centipede Game.^26^^,^^27^ The Centipede Game is a sequential dilemma that captures questions related to trust, competition, cooperation, and risk-taking, allowing one to gain insight into moral preferences of its participants.^28^ While there exist several variations of this game,^29^ we will base our discussion on the Incremental Centipede Game (ICG), considering at a later stage how switching to the Constant-size Centipede Game (CCG) influences the selection for lower or higher ToM levels (see STAR methods and supplemental information for details on the CCG), covering in this way a variety of situations wherein ToM may be considered relevant. From an ecological perspective, the Centipede Game can be considered a sequential common-pool resource game,^30^ where each participant has exclusive access to consume the largest fraction at different moments. Different from the CCG, the resource grows in the ICG, tempting consumers to wait for a larger share, yet running the risk that the other gets the most when accessing it.

**Figure 1:**
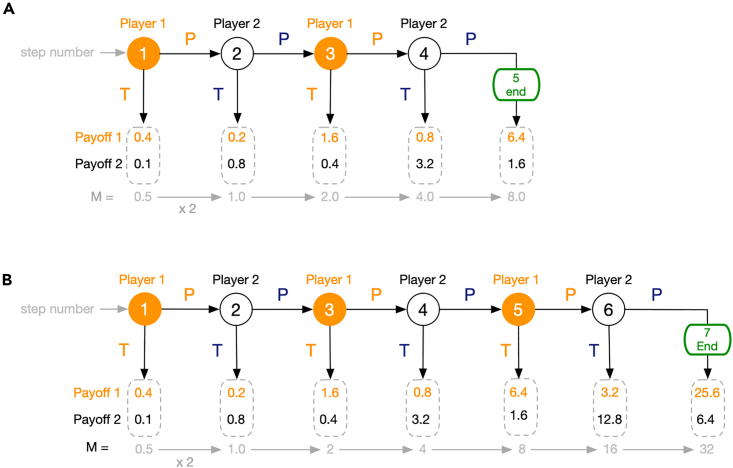
Defining the Incremental Centipede Game (ICG) (A) The ICG as in^31^ with L=4. The game starts with a resource M=0.5 that doubles in every step of the game. Each Player - 1 in odd steps (orange) and 2 in even steps (black) - must either play Take (T) or Postpone (P). Playing T in step l∈[1,4] means ending the game and receiving 80% of the resource available (here $2^{l-1}M$). Playing P means doubling the value of the resource and letting the other Player decide what to do in the next step. Whenever Player 2 plays P in step l=4, a final doubling of the available resource takes place, the resulting amount being split with 80% given to Player 1. Payoffs of Player 1 (2) at each possible step are shown in orange (black). Players will employ conditional strategies defined by t∈[1,5], where t represents the lowest possible step at which to play T (t=1, means always-T whereas t=5 means always-P). (B) The L=6 ICG.^31^ The game proceeds in exactly the same manner as in A except that the total number of Steps is now L=6.

The ICG is a game with perfect information wherein interdependent reasoning has been considered important and participants experience uncertainty about the motivations of the co-players, producing thus unexpected outcomes, a requirement for the study of ToM.^2^ Concretely, the ICG involves two individuals that take turns (in a total of L steps) deciding between two actions (T = Take, P=Postpone) regarding the split of a resource of initial value M that grows at each Step l (l∈[1,L]) of the game. Player 1 (Player 2) plays at odd (even) steps. Playing T in Step l means ending the game and receiving the biggest part of the resource available. Playing P means growing linearly or exponentially the value of the resource and letting the other Player decide what to do in the next step. Whenever Player 2 plays P in step l=L, different outcomes may be considered.^29^ Here, a final growth of the available resource takes place, the resulting amount being split in favor of Player 1. The game is illustrated in Figure 1, where game structure and payoff values were adopted from McKelvey and Palfrey’s behavioral experiments^31^ for L=4 and L=6.

Backward induction in the ICG leads a fully rational *Homo Economicus* to play T in step l=1, a feature that experimental results repeatedly contradict^29^^,^^31^: The majority of experiments end between 1/3^rd^ and 2/3^rd^ of the number of steps in the game.^29^ Only when a specific combination of strong conditions (e.g., very high stakes and costly P moves, or payoff asymmetries) occurs participants will end the game in the first round. From an evolutionary perspective, in a world where individuals have no ToM and simply play T at pre-defined steps and the population changes by replacing less successful strategies with more fit ones, natural selection (if sufficiently strong) also leads to a scenario in which most individuals end up playing T in step l=1.^32^^,^^33^ Weak selection, associated with stochasticity in the evolutionary process in finite populations, has been invoked to explain the differences between experimental observations^31^ and the expected rational outcome. Yet, this argument ignores that human strategies involve interdependent thought processes, which are, as argued earlier, crucial for real-world social interactions and decision-making. We therefore ask the question: What if individuals may evolve a ToM? And, if so, will ToM prevail under natural selection? How will individuals endowed with a ToM behave in the ICG?

Here we show how individuals with a ToM evolve and dominate in a population interacting via the ICG. By requiring a best fit of our model results to available behavioral experimental data, we observe that evolution leads to a population where boundedly rational individuals prevail. Importantly, our model also exhibits, in an unforeseen manner, the co-evolution of a ToM together with an optimism (or *positive illusion*) about the behavior of others as well as the emergence of new equilibrium configurations in the ICG (in close agreement with experimental observations) that deviate from the classic rational equilibrium of taking at the start: ToM strategies appear to change the pure dominance of rational strategies by introducing a coordination game structure^34^ into the ICG, which provides a basin of attraction toward new equilibrium configurations where optimistic beliefs about others’ behavior and bounded levels of reasoning coexist, and which results in taking halfway through the game. Our results remain robust under different conditions and switching to a dilemma with less temptation for higher future rewards, i.e., the CCG, leads to a redistribution of ToM strategies, favoring strategies with neutral beliefs and low (or even no) reasoning capacity, as has also been experimentally observed.

### Introducing theory of mind as a strategy

We equip individuals with a ToM strategy of variable cognitive capacity, associated with a parameter k≥0.^35^^,^^36^ Strategies are thus not only defined by a parameter t, indicating the *prior belief* about the earliest possible Step at which to play T in both roles as in,^32^^,^^33^ but also the number of iterative reasoning steps that will determine how to act given the personal beliefs. Together they are thus represented by a duple (t,k), which will be used to infer what to do in the roles of Player 1 and Player 2 in the ICG.

**Figure 2:**
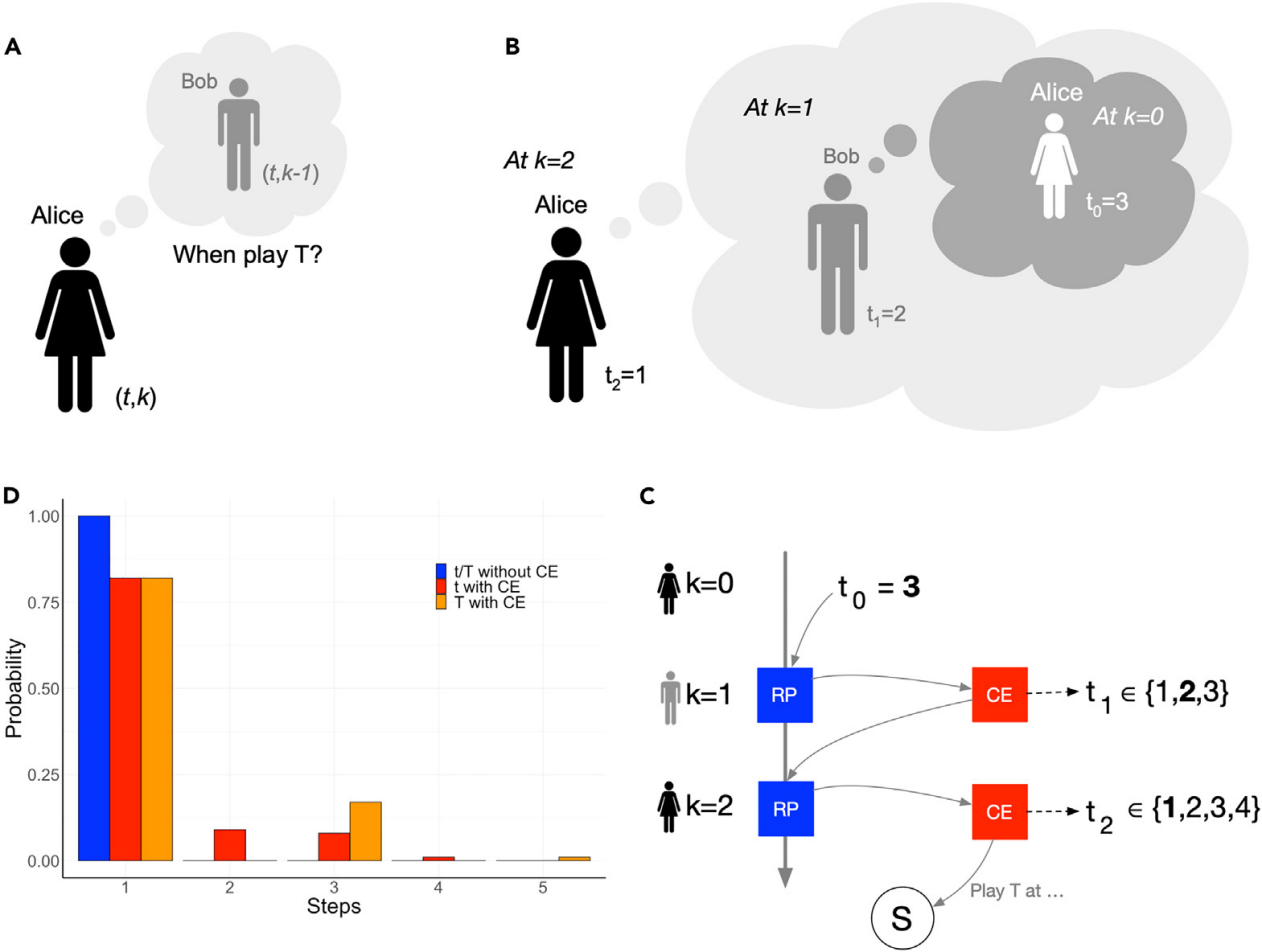
Illustrating the workings of ToM strategies (A) Strategies are defined by a duple (t,k), with t being the earliest moment of playing T and k the recursive reasoning capacity of the individual. Given Alice’s strategy (t,k), she believes her co-player Bob will use strategy (t,k−1). Under this belief, she will reason upon how Bob will respond to her moves and how she will respond to those hypothetical moves from Bob. (B) The recursive RP of Alice with ToM strategy (3,2), acting as Player 1: To know $t_{2}$ at k=2, Alice needs to infer what she believes Bob will do at k=1 ($t_{1}$) given what he believes about what she would do at k=0 ($t_{0}$). In this example Bob will respond to Alice’s $t_{0}=3$, which will lead him (according to Alice’s RP) to take as soon as Step 2 ($t_{1}=2$); Given this result, she will respond by taking as early as Step 1 ($t_{2}=1$). Several RP variations can be conceived; the present example mimics deterministic best response to the beliefs one has about the co-player. Three recursive RP are considered as examples in this paper as discussed in Figures S1–S3, with the focus on Figure S3, as it provides the best match with the experimental data. (C) While in panel B no errors are made in the RP, here cognitive errors occur with a probability defined by a parameter ε. The “no error” choice x will happen with probability 1−ε, whereas with probability ε the choice will be to play T at x±1. Combined with a recursice RP, the cognitive errors will lead to a branching process which produces a probability distribution over t (a mixed strategy), illustrated in the next panel. (D) Without cognitive errors (ε=0), the RP will lead the ToM strategy (3,2) to always try to take as early as Step 1 (blue). With cognitive errors (ε>0) it becomes possible to take at Step 2, 3 and 4 (red), albeit with very low probabilities. As Player 1, Alice can only play T at Steps 1, 3 and 5. The orange bars show the probabilities of playing T given the distribution over t.

At the lowest level, k=0, no ToM is present, and individuals simply play T as soon as possible for the given t: For instance, an individual with t=3, will play T at Step 3 (Step 4) as Player 1 (Player 2). Individuals with ToM strategies (t,k) (with k>0) believe that their opponent will employ the strategy (t,k−1) (see Figure 2A) and, as a result, will compute a response to such an (anticipated) behavior, where we use here an RP similar to a level-k hierarchical recursive model.^35^^,^^37^ As illustrated in Figure 2B, Alice, who has an initial belief t=3 and a capacity to reason up to two recursive steps (k=2), needs to find a response to what she believes any other individual, that she interacts with via the ICG, will do. She assumes that these individuals, which she mentally refers to as Bob, have the same initial belief t=3 but can only reason up to one step lower in the recursion, i.e., k=1. As she believes that Bob uses the same RP as her, she may infer that he will decide to take as early as one step before the moment he believes she would take, i.e., he may decide to take as early as Step 2 ($t_{1}=2$) given that he thinks she has no reasoning capacity (k=0) and that she will thus try to take as early as Step 3 ($t_{0}=3$). Given this RP inference, Alice may choose to take even one step earlier ($t_{2}=1$) using her RP. She thus reasons that her best choice is to take as early as Step 1 and she will use this action in the interaction with the other individuals she encounters. Note again the difference with prior work,^23^^,^^24^ where strategies differ in how they acquire through inference or learning (or not) insights into some hidden mental state of a co-player, which is not the question being researched here.

By making the belief part of the strategy and inferring the action from the belief, our model no longer assumes that individuals need to hold the same beliefs as the co-players or even have correct beliefs^38^^,^^39^: Each individual in the current evolutionary model starts from a private prior belief (or type) on what the other may do and selects a (noisy) response to that based in the RP. An important consequence will be that the decision (by individuals with a ToM) at which step to play T may coincide (or not) with their *a-priori* belief (encoded in their strategy as t), a feature which cannot happen in the absence of a ToM.

We further allow individuals to be boundedly rational,^40^ so that cognitive errors in computing the response may occur with a probability ε≥0: whenever ε>0, individuals may decide to Play T at one step higher or lower (at each k-level) than what the RP computation would determine.^35^^,^^37^ For simplicity ε is assumed to remain constant in the population, leading to a branching process in the propagation of errors. Figure 2C shows that in Alice’s RP the cognitive errors introduce the possibility to deviate from what the correct response should be: Instead of arriving at the conclusion that every co-player in the ICG will take as early as Step 2 at k=1 ($t_{1}=2$), she may arrive at the conclusion that they will take as early as Step 1 or 3 (so $t_{1}=1$ or $t_{1}=3$, each with a probability $\frac{\varepsilon }{2}$). The implication is that at the next reasoning level she would infer a new response based on that cognitive error, propagating the error to higher reasoning levels. Together with any additional cognitive errors, this may lead her to decide to act as early as $t_{2}\in \left\{1,2,3,4\right\}$ at the next level.

Multiple executions of this stochastic RP will thus produce a probability distribution over the actions that an individual can take given her initial beliefs and predefined maximum k-level, as can be seen in Figure 2D: Whereas no cognitive errors always leads the individual to take as early as Step 1 (blue bar), the occurrence of cognitive errors leads to a probability distribution of playing at any Step (red bars). Each ToM strategy thus samples its own probability distribution to determine how to interact with the other strategies in the ICG. Note that, while Alice can choose to act as early as any Step, she can act only at specific Steps when being Player 1 (or Player 2): the t-distribution thus maps into a T-distributon, which specifies the actual likelihood of acting in a specific role in the ICG (mapping from red to orange bars in Figure 2D).

Different implementations for the RP can be considered, each leading to different t-distributions, that for some RP may even be determined analytically. Figures S1–S3 provide a detailed description of those used in the current article. The focus in the remainder of the text will be on the *inertia* RP discussed in Figure S3 as this provided the best fit to the experimental data. We will return to the two alternatives presented in Figures S1 and S2 (see also STAR methods) later in the article. We do not consider an evolutionary competition between different RP here, as each individual is using the same RP, albeit with different sophistication as given by k.

In STAR methods we provide furthermore details on how individuals, equipped with these (probabilistic) ToM strategies interact and evolve in a finite population,^41^^,^^42^ where individual fitness is computed as the average payoff obtained by interacting with many other (randomly chosen) individuals in the population, where in each interaction we compute the payoff obtained by acting both as Player 1 and as Player 2. Successful (t,k) strategies are thus more likely to survive when beliefs and reasoning prowess lead to good decisions in both roles of the ICG.

## Results and discussion

**Figure 3:**
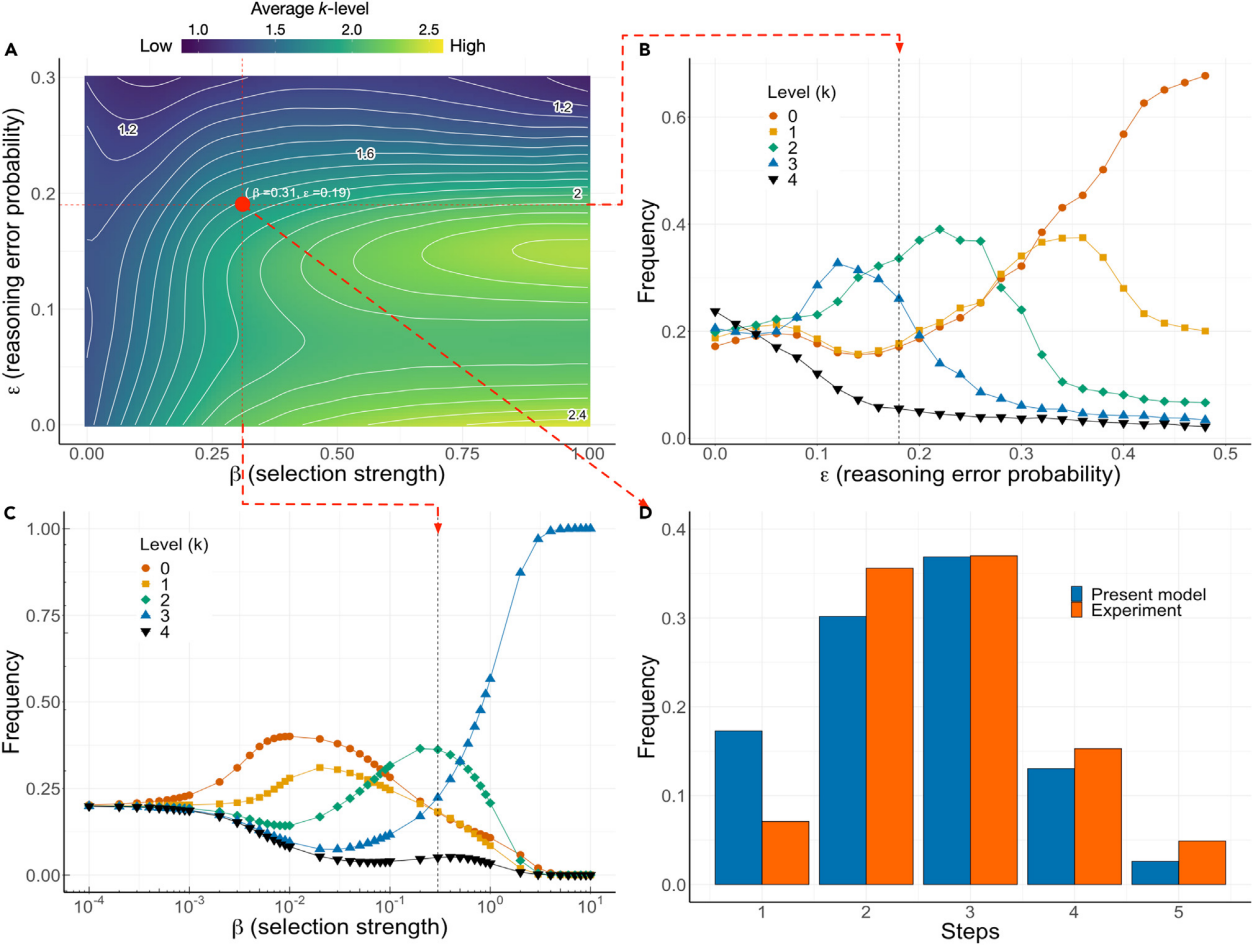
Evolution of a ToM (A) Average k-level emerging from evolving a population of Z=500 individuals as a function of the selection pressure β and the cognition error probability ε. Fitting our stationary distribution of Steps to those deduced from behavioral experiments leads to the optimum values ($\beta^{*}\approx 0.31,\varepsilon^{*}\approx 0.19$) depicted with a red circle in panel A. This panel shows how the average recursive reasoning level k changes in the population in the function of β and ε. Comparison between behavioral experiments and the theoretical best fit is made in panel D. Panels B and C portray the k-level distributions as a function of ε and β, respectively, in each case keeping the other parameter at the optimum value ($\beta^{*}$ and $\varepsilon^{*}$, respectively). This result shows how ToM, specified here as a process of recursively reasoning about beliefs and k-levels of others, evolves, illustrating how both parameters influence the selection for lower or higher recursive reasoning levels.

Figure 3 shows the results of our model after letting all 25 (t,k) strategies, with t∈{1,2,3,4,5} and k∈{0,1,2,3,4}, evolve in a finite population in the ICG with L=4, for different values of the free external parameters of the model: selection pressure β and cognitive error probability ε (see introduction and STAR methods). In Figure 3A we plot the average k-level, where the red circle indicates the optimum combination ($\beta^{*}\text{,}$ $\varepsilon^{*}$) at which a best fit to the data from behavioral experiments^31^ is obtained (see direct comparison with behavioral experiments^31^ in Figures 3D and S4). Figures 3B and 3C portray the dependence of our results for the k-level distribution on each of the parameters (β,ε) while keeping the other fixed at the optimum value. Whereas increasing ε (at $\beta =\beta^{*}$) selects for low k-levels, increasing β (at $\varepsilon =\varepsilon^{*}$) appears to have the opposite effect.

The results in Figure 3 show that ToM evolves, i.e., we obtain the emergence and prevalence of strategies with k>0 for a wide range of values of the external parameters β and ε, providing a novel view of the evolution and role of strategies including a ToM in finite populations (see also Figure S5). At the best fit values to the behavioral experiment data, evolution leads to populations exhibiting a distribution of cognitive capacities where boundedly rational^40^^,^^43^ individuals, i.e., those with limited k and ε>0, prevail (in accordance with experimental studies of ToM^31^ and their analyses^44^). The model thus predicts that, for a wide range of β and ε, evolution leads to populations exhibiting a heterogeneous distribution of cognitive capacities where low to intermediate values of cognition prevail, as shown in Figure 3, panels B and C. Indeed, only when ε≅0 and/or β≅0 are all levels of cognition and beliefs equally likely. Equivalent observations can be made for the ICG with L=6, as shown in Figures S6 and S7.

**Figure 4:**
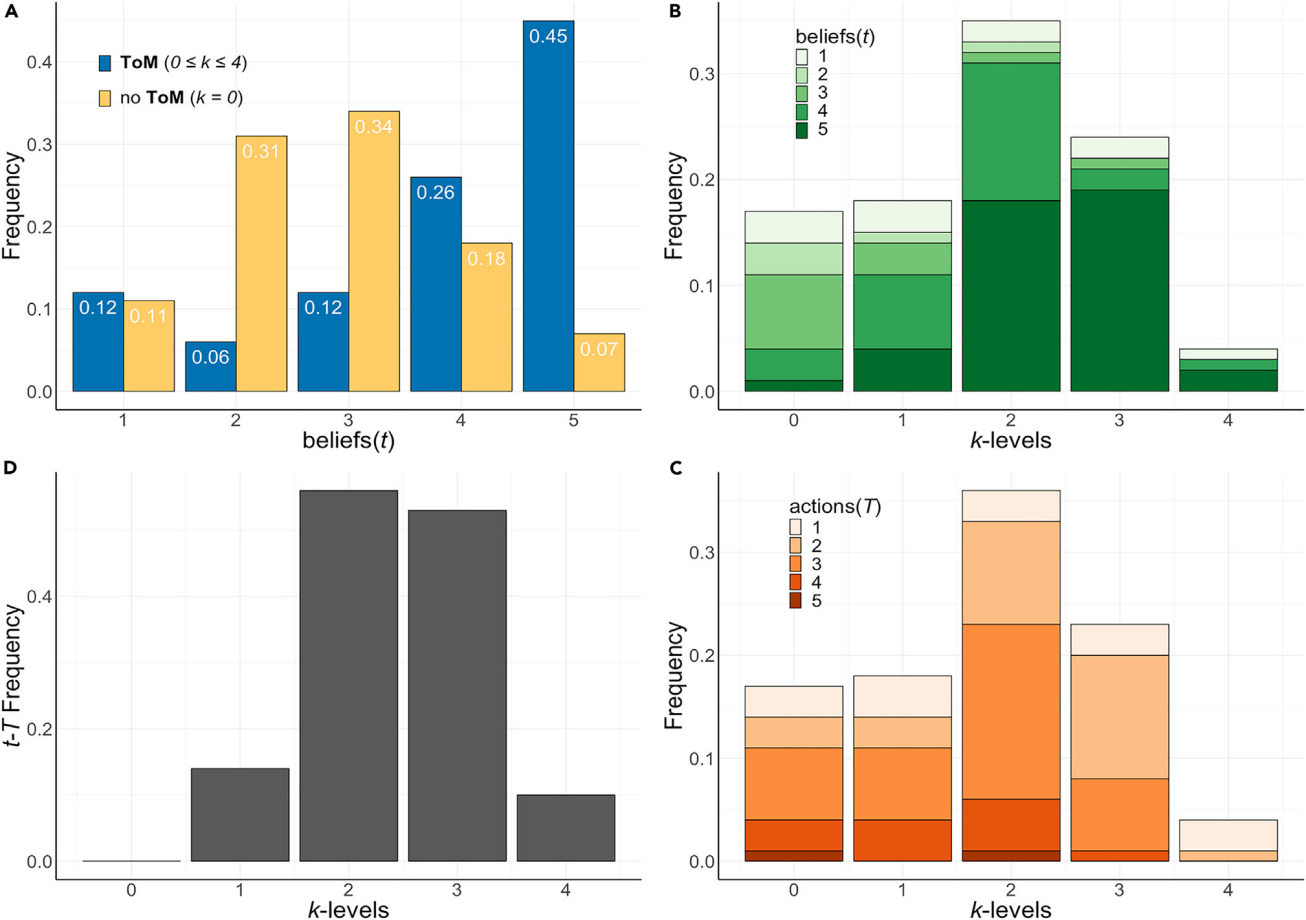
Evolution of beliefs and reasoning levels for the calibrated model (A) Direct comparison between the evolution of the beliefs in the absence (k=0) and presence (0≤k≤4) of a ToM. Strategies incorporating a ToM (k>0) evolve higher beliefs than otherwise. This panels reveals that when ToM is added as a strategy, beliefs become skewed toward higher t values, corresponding to an optimistic perspective about the actions of other players in the ICG. (B) Composition of the population as a function of k-level; for each k-level, the distribution of beliefs (t) is shown. Individuals adopting strategies with a ToM (k>0) prevail in the population, where intermediate values of k dominate and coexist with high beliefs. One can see that different levels of recursive reasoning co-exist for this combination of β and ε, and that higher k-levels are more likely to have optimistic beliefs (C). Same as (B) except that now, for each k-level, the distribution of actions (T) is shown in the context of the k-levels. (D) This panel compares explicitly the actions and the beliefs associated with each k-level. As can be observed, only in strategies with no ToM (k=0) there is no mismatch between actions (T) and beliefs (t); for all other strategies there is a significant amount of mismatch, reflecting the co-evolution of an optimism bias together with a ToM. In this figure Z=500, $\beta^{*}=0.31$ and $\varepsilon^{*}=0.19$.

Figure 4 provides details of another important feature of our model–related to the co-evolution of (mis)-beliefs and ToM. To this end, we provide detailed information about the belief (t), reasoning (k) and action (T) distributions in the population at the best fit parameter values ($\beta^{*},\varepsilon^{*}$). In Figure 4A we show how the distribution of beliefs changes depending on whether individuals may or may not evolve a ToM: allowing k>0 (blue) leads to the emergence of an optimism bias (t>T), as the distribution becomes skewed toward high t– values compared to the limit k=0 (yellow), a feature that naturally stems from the prevalence of individuals with k>0, as their actions (T– value, Figure 4C) mostly take place at values smaller than their beliefs (t– value, Figure 4B) as shown also in Figure 4D. Note that this optimism bias focusses on beliefs about others, thus differing from prior work where individuals are optimistic about themselves and the gains from their own actions.^45^^,^^46^^,^^47^ In line with what was observed in Figure 3, the reasoning levels that co-evolve with such an optimism bias do not peak at k=4: Instead, the distribution of k-levels peaks at k=2 and strategies with k>3 become the least prevalent in the population. This inherent limit on the k-levels, appearing without any form of reasoning cost, is even more apparent in the 6-Step ICG, as is visualized in Figure S8, where strategies with k>3 are either marginally present or totally absent in the stationary distribution, even when they were allowed to go up to k=6. Our results thus reveal that the individual reasoning capacities remain limited in the competitive context provided by the ICG, even under stronger selection strengths (high β).

Not only do the results visualized in Figure 4 remain robust for larger ICG. Also, for different population sizes Z (provided Z is not so small that finite size effects dominate, Figure S9), boundedly rational ToM strategies and misbeliefs emerge consistently. Numerical simulations confirm the analytical results, indicating that they also remain valid for sizable mutation probabilities (Figure S10), here modeling situations in which individuals spontaneously replace their strategy by a randomly chosen one. Additionally, even when transitions between strategies with different k-levels are inhibited (compared to transitions between strategies with different beliefs)–this way mimicking the idea that beliefs are more likely to change than one’s own cognitive capacity–the results remain robust, as shown in Figure S11. Finally, introducing a simple linear cost associated with higher k-levels will change the overall belief patterns only when the cost added to each additional reasoning level becomes excessive (Figure S12). Nonetheless, misbeliefs remain prevalent in the ICG even under those conditions.

The complexity of the co-evolutionary dynamics of beliefs (t) and k-levels leading to the emergence of an optimism bias in individuals endowed with ToM is best understood at high selection pressure (for details, see Figure S13). In this regime, we observe the emergence of a new equilibrium associated with the tuple (5,3) which becomes *Evolutionary Robust*^48^^,^^49^ (i.e., all outgoing edges are either 0 or have a fixation probability lower than neutral fixation, as confirmed numerically). This (5,3) strategy, unanticipated by backward induction and not previously identified,^21^^,^^32^^,^^33^ strongly correlates with the experimental observations.^31^ Indeed, and contrary to the evolutionary dynamics at high β in the absence of a ToM, where low values of t are selected, boundedly rational individuals ($\varepsilon^{*}=0.19$) endowed with a ToM reflecting intermediate reasoning capacities are now able to sustain high (mis)beliefs at t=5.

**Figure 5:**
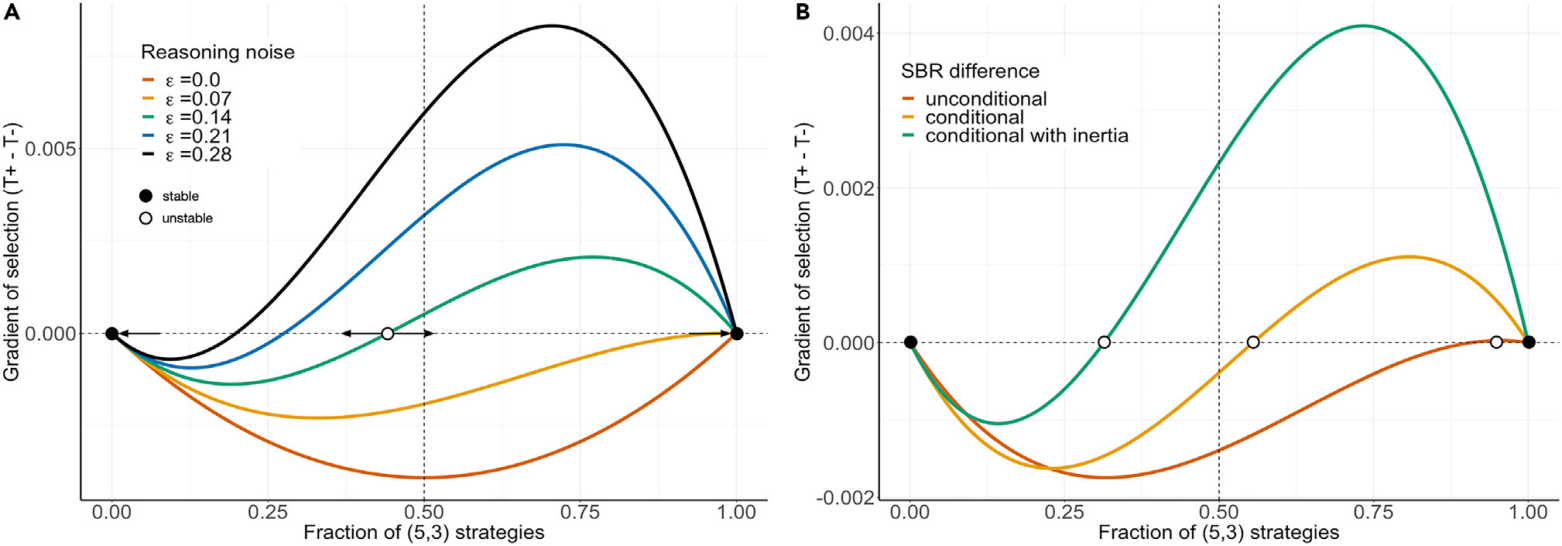
**ToM generates a coordination dynamic between (**1,0**) and (**5,3) (A) Gradient of selection^75^ for different values of ε in a population where individuals may adopt only the 2 strategies (1,0) and (5,3). Whenever ε≤0.06, rational choice–the strategy (1,0)–constitutes the only attractor of the dynamics. Whenever 0.06<ε, the finite population analogue of an unstable fixed point emerges, leading to the appearance of a coordination dynamics and a basin of attraction toward (5,3) which increases with increasing ε. Open and closed circles represent the finite population analogues of unstable (repeller) and stable (attractor) fixed points, respectively. This image shows that introducing ToM as a stochastic recursive RP based on the beliefs about the behavior of others transforms the ICG game, allowing for the emergence of a new evolutionary robust strategy. (B) This panel shows moreover that the results depend on the type of RP used (see STAR methods). Three types of RP mechanisms are considered, i) an *unconditional* RP that makes the player decide to always take one step before when she believes the co-player will take (always at t−1), ii) a payoff-*conditional* RP that compares the personal payoffs at the co-players t and t−1, switching to the most beneficial one and iii) the payoff-conditional RP with *reasoning inertia* discussed in Figure 2, conditionally switching to t−1 or continue with what was inferred at the earlier reasoning level. While these RP are not exact copies of human cognitive processes, it shows nonetheless that variations in RP will affect the outcome, potentially opening the door to unanticipated equilibrium behavior. As can be observed here, the evolutionary robust strategy (5,3) becomes more likely through a combination of payoff comparison and reasoning inertia. In this figure Z=500 and $\beta^{*}=0.31$. In panel B, $\varepsilon^{*}=0.19$.

At the optimum selection pressure ($\beta^{*}=0.31$) this equilibrium configuration is still prevalent and undergoes a coordination type dynamic with the rational equilibrium, as shown in Figure 5A), where the evolutionary dynamics is studied along the path connecting these 2 configurations. The gradients of selection (see STAR methods) of Figure 5A shown for different values of the reasoning error ε, indicate that, for low ε (ε≤0.12), selection favors a population of rational players (either by being dominant (ε≤0.06) or by having the largest basin of attraction (0.06<ε≤0.12)). For ε>0.12 however, evolution favors strategy (5,3), whose basin of attraction grows with increasing ε. Also, in the ICG with L=6, the well-known (1,0) equilibrium is replaced by a strategy with optimistic beliefs and bounded rationality; In that case, we see that now t=6 with k=2 becomes Evolutionary Robust once β>2 (Figure S6C).

The type of RP that is employed ultimately defines the location of the coordination point (minimal fraction of (5,3) strategists needed in the population to move into this new equilibrium, as shown Figure 5B) as well as in the overall predictions of the model: Whereas the inertia RP kernel prioritizes the importance of k=2 and k=3 when considering the optimal $\beta^{*}$ and $\varepsilon^{*}$ that fit the behavioral experiment data, the two other models (the unconditional RP and payoff-conditional RP detailed respectively in Figures S1 and S2) show a preference for k=0 and k=1 to explain the data, albeit to a different extent, as can be observed in Figure S14. Moreover, for selection strength $\beta \gg \beta^{*}$, the inertia RP leads toward a monomorphic population of (5,3) strategies (Figure 3C). In case of the two other RP, high β will lead to a population consisting only of the (1,0) strategy (considering their optimal $\varepsilon^{*}$). Relying thus on previous inferences as in inertia RP, provides an important road toward the prevalence of the new equilibrium in both the L=4 and L=6 ICG.

While the strategic situation captured by the ICG provides a relevant scenario for investigating the evolution of a ToM,^2^ other games, even within the class of centipede games, may not be as conducive to higher ToM or to actions that do not correspond to the known subgame-perfect equilibrium of the centipede game. In the CCG^29^^,^^50^ (for details, see Figures S15–S19), both ToM and belief types are skewed toward the lower values (with average beliefs close to the minimum), thereby producing actions aligned with the beliefs, removing thus the optimism bias that was observed in the ICG. While strategies with higher k-levels with significant cognitive errors survive (at rapidly decreasing frequencies), strategies with k=0 and small t dominate in the fitting between the model and the experimental CCG results,^50^ producing behavior that corresponds to taking as early as possible. Also, the higher k-levels that can be observed are associated with small t values, similarly, leading to taking as early as possible. Our analysis thus reveals that the temptation for higher future rewards, as in the ICG, is a determining factor for bounded higher-order ToM and associated misbeliefs, and thus taking the risk of playing T later in the game, to evolve, a property that seems to correlate well with the general findings of prospect theory.^51^ The differences observed here between ICG and CCG align with recent experimental work that examined through belief elicitation experiments the differences in rationality and beliefs in rationality between ICG and CCG.^52^ Significant higher belief in rationality and higher-order beliefs in rationality were seen in the CCG, corresponding to the more rational strategy distribution (and the lack of misbeliefs) produced by the evolutionary model.

The empirical results presented here may also have implications for the study of correlated equilibria^53^ and how to reach them through simple learning dynamics.^54^^,^^55^^,^^56^ Notwithstanding the need of further formal verification, observations in Epistemic Game Theory^57^ support the hypothesis that the new equilibria identified here for the ICG may be correlated equilibria: It has been shown that individual actions may become correlated when there exist correlations in the players’ beliefs and higher order beliefs about how the game is played.^58^^,^^59^ The resulting correlation is called intrinsic as there is no extrinsic signal or mediator to introduce the correlation as in the original work by Aumann.^53^ In our ToM evolutionary model, all individuals use identical beliefs for both roles in the ICG, assume the beliefs of the co-player are the same as their own and they all use the same RP to reason about their beliefs. These assumptions may be sufficient to produce correlated beliefs and actions, suggesting thus that the new equilibria where individuals have optimistic beliefs and have bounded reasoning capacity are in fact correlated equilibria of the ICG. Our results thus provide novel insights that expand previous research into whether such equilibria can be found via evolutionary processes,^60^^,^^61^^,^^62^ with the novelty following from the observation that the new results are based on intrinsic correlation processes as opposed to extrinsic ones.

While current ICG results focused on one-shot sequential interactions, extensions can be imagined to repeated sequential as well as non-sequential interactions. In that context, a deeper analysis on schemes to revise the initial beliefs (e.g., based on some form of Bayesian belief-updating mechanism) or schemes to better identify the k-levels of the others become possible,^22^^,^^23^^,^^24^^,^^25^^,^^63^ providing also the potential to generate hypothesis for future experimental work on decision-making and belief elicitation.^38^^,^^39^^,^^52^

Similar to prior work in evolutionary game theory, this work also demonstrates the importance of noise in relation to strategic decision-making (see for instance^64^^,^^65^^,^^66^), leading potentially to outcomes that deviate from known equilibria. Further research will need to be carried out to investigate how the current parametric cognitive noise (expressed as ε) may be translated to actual human thought processes, which are known to be noisy,^67^ in order to come one step closer to explaining the centipede game, and consequently also other strategic games.

To conclude, we showed under different conditions that ToM co-evolves with misbeliefs in the ICG and that the resulting strategies are boundedly rational, i.e., they are limited in their capacity to reason (k-level) and to do this correctly (ε>0), even without the introduction of reasoning costs. The main observation is that introducing ToM strategies in the population transforms the competitive nature of the ICG in such a way that alternative, potentially correlated, equilibria, which translate into behavior that better resembles human behavior, emerge. The likelihood of reaching these new equilibria depends in part on the sophistication of the RP, with certain methods introducing stronger deviations from the standard game theoretical results. These observations are independently supported by psychology research on optimism bias in the human species,^68^^,^^69^ evolutionary research on the adaptiveness of human biases^70^ and the importance of self-deception^71^^,^^72^ and reality-denial,^73^^,^^74^ where the latter has been suggested to explain why humans may have been the only species to reach the level of intelligence that appears to distinguish them from other animals on the planet. Together, our results introduce novel insights into the effects of ToM on decision-making in strategic situations, opening a door to new routes for competitive and cooperative theories on the origins of ToM.

### Limitations of the study

ToM as it can be observed in humans nowadays is the result of an evolutionary process where a variety of challenges and social environments were experienced over a long timescale. It encompasses a multi-faceted collection of capabilities, both cognitive and affective, the evolution of which cannot be easily disentangled. A single model using a specific set of assumptions and social (game) structures, will therefore be unable to capture the plethora of different challenges and social environments that ultimately evolved the ToM that we can identify at present.^21^^,^^22^^,^^23^^,^^24^ Notwithstanding the novel insights that were obtained with the current model, there are thus limitations due to the assumptions made, namely, that individuals have fixed prior beliefs about their co-players, that they believe that everyone has the same beliefs as themselves, that they all use the same reasoning process, and they believe others are less smart in terms of reasoning. Moreover, only one class of sequential game structures, albeit general enough to investigate the questions that were asked,^2^ was investigated here. Clearly future work will surely relax some of the assumptions made here to see whether the results remain consistent. At the same time, additional complexities (e.g., repeated interactions, variable physical and/or social environments, and so forth) may be taken into consideration to further explore their impact on the evolution of ToM. We have shown, so far, that our main results remain consistent under a series of perturbations, supporting thus their interest and generality for further study.

## STAR★Methods

### Key resources table

**Table:** 

REAGENT or RESOURCE	SOURCE	IDENTIFIER
**Software and algorithms**		

Stochastic Evolutionary Dynamic Code for Theory of Mind in the Incremental Centipede Game, including data and R scripts for figures.	https://github.com/tlenaert/ctpToMstoch.git	https://zenodo.org/records/10230142
Moran simulation code for Theory of Mind in the Incremental Centipede Game, including data and R scripts for figures.	https://github.com/tlenaert/ctpToMmoran.git	https://zenodo.org/records/10230225
Stochastic Evolutionary Dynamic Code for Theory of Mind in the Constant-size Centipede Game	https://github.com/tlenaert/cpieTomStoch	https://zenodo.org/records/10451365

rowhead**Other**		

Data from, R.D. McKelvey and T.R. Palfrey (1992) An Experimental Study of the Centipede Game	Econometrica 60(4):803-836	https://doi.org/10.2307/2951567
Data from, Fey, M., McKelvey, R. D., & Palfrey, T. R. (1996). An experimental study of constant-sum centipede games.	International Journal of Game Theory 25:269-287.	https://doi.org/10.1007/BF02425258

### Resource availability

#### Lead contact

Further information and any related requests should be directed to and will be fulfilled by the lead contact, Tom Lenaerts (Tom.Lenaerts@ulb.be).

#### Materials availability

This study did not generate new unique reagents.

#### Data and code availability

• All data and scripts used to produce the figures in this paper have been deposited on GitHub and Zenodo, and are publicly available. The DOIs are listed in the key resources table.
• All original code that produced the data is deposited on GitHub and Zenodo, and are publicly available. The DOIs is listed in the key resources table.

### Method details

In the following sections the different elements needed to implement the model and produce the results are explained. The code for the ToM evolutionary model as well as the scripts for analysis and the data for reproducing the figures are available for research on Zenodo.^76^^,^^77^

#### Centipede game

The Centipede Game was introduced in 1986 by Rosenthal.^26^^,^^27^ While the manuscript results focus on analysing an Incremental Centipede Game (ICG), more specifically an ICG with an exponentially growing resource, there exist several variations of this game.^29^ One such class of variations can be found in the payoff structure, i.e., the joint payoff over the different steps can remain the same, grow linearly or grow exponentially. Another variation is how the game ends, i.e., the final step can either have an equal or unequal division of the resource (favoring one of the participants as in our case), or even a zero payoff for both (called centipede game with a deadline). Figure 1 illustrates the exponentially growing ICG for L=4 and L=6. The equal division ICG scenario only modifies the last step as is illustrated in Figure S15A. The Constant-size Centipede Game (CCG)^50^ differs from the ICG in that the resource M remains constant at each Step l (l∈[1,L]) of the game, only the split changes: Playing T in the first step results in an equal split of M between both participants, while playing T in any step l>1 means ending the game and receiving an increasingly larger share of the resource. Playing P means changing the split of the resource and letting the other Player decide what to do in the next step. Whenever Player 2 plays P in step l=L, a final division favoring Player 1 is proposed. The game is illustrated in Figure S15B, where game structure and payoff values were adopted from the Fey et al. behavioral experiment^50^ for L=6. Figures S16–S19 in the Supplemental Information show the results for the CCG equivalent to those shown for the ICG in the manuscript.

#### Stochastic reasoning processes

An individual derives her actions from her personal beliefs using an RP. Figure 2 in the main manuscript provides an abstract view on how such RP work recursively, showing also how cognitive errors produce a probability distribution over the actions. As every individual will act as Player 1 and Player 2 in the ICG, the action inference needs to be performed for both roles. Figures S1–S3, show in detail three possible RP, among others, that may be used for the recursive inference. In Figure S1, the RP assumes that at each step of the reasoning process the individual will always switch to one step before the moment when she believes the other individual is going to take. So, if she believes the other will take as early as t, she will switch to t−1. We refer to this as the *unconditional* RP. In Figure S2, an individual determines a best response to what she believes the co-player will do: She compares the payoff she would receive in her role given the beliefs she has about t and t−1 of the co-player. She will select the one that gives her the highest payoff in her role. This RP is called the (payoff-) *conditional* RP. Finally, in Figure S3, *inertia* is added on top of the conditional RP. In this RP, Alice decides to act one step before the co-player if that gives her a payoff benefit compared to taking at the same time as the co-player. If there is no payoff gain, she will continue to take as early as what she inferred before, at lower k-levels. So, theoretically, she might choose to take as early as her initial belief at k=0 when she sees no payoff advantage to take before her imagined co-player at any next step in the recursive RP. We refer to this RP as the inertia RP.

#### Fitness calculation

The Payoffs in the ICG for L=4 (L=6) are shown in Figure 1. From this payoff structure, the fitness $f_{X,Y}$ of an individual X when interacting with any individual Y is numerically computed by playing the ICG in both roles. Given the stochastic nature of the reasoning process, the interaction between a pair of individuals is repeated multiple times to arrive at an average behavior over all possible actions. At each repetition both individuals infer first their actions T as Player 1 and Player 2 and then the payoff of their interaction in both roles is calculated. Concretely, a Monte Carlo sampling is used to obtain an average over multiple interactions wherein the inferred actions come from a distribution over the possible actions induced by ε. This average can be expressed thus as follows:

$$f_{X,Y}\left(\varepsilon ,R\right)=\frac{1}{R}\sum_{r=0}^{R}\pi_{r}\left(SRP\left(t_{X},k_{X},\varepsilon \right),SRP\left(t_{Y},k_{Y},\varepsilon \right)\right)$$

with R the number of steps in the Monte Carlo sampling, SRP(.) one of the stochastic RP functions discussed earlier and $\pi_{r}\left(.\right)$ the average payoff at Monte Carlo step r obtained for acting in both roles, i.e., X acting as Player 1 as well as Player 2 in the ICG (see Figure 1). Throughout the paper $R=5\times 10^{4}$ was used to have a good estimate of the individual fitness values.

#### Evolutionary dynamics

We consider a well-mixed population of finite size Z, where individuals may use any of the strategies associated with the duple (t,k), with a total of ${\left(L+1\right)}^{2}$ different strategies (25 for L=4 and 49 for the L=6). Strategies evolve according to a mutation-selection process defined in discrete time. At each time step, the strategy of one randomly selected individual X is updated. With probability μ, X undergoes a mutation. In that case, X adopts a strategy drawn randomly from the available strategies. With probability 1−μ, another randomly selected individual Y acts as a potential role model and will be imitated by X with probability^78^ $p={\left(1+e^{-\beta \left(f_{Y}-f_{X}\right)}\right)}^{-1}$ that increases with the fitness difference between *Y* and *X*. The parameter β represents (in the population of size Z) the intensity of natural selection or the strength of imitation: when β=0, imitation occurs with 50% chance, corresponding to the process of neutral drift. When β is large, imitation becomes sensitive to the slightest fitness difference, rendering evolution almost deterministic.

In the limit in which mutations are rare, we can compute analytically the prevalence of each strategy.^79^ In this limit, the time interval between two mutations is sufficiently large that evolution will lead to the fixation of one strategy in the population before the next mutation leads to the appearance of a new strategy. Thus, at any time, there will be at most two strategies simultaneously present in the population.^80^^,^^81^ This rare mutation limit leads to an embedded Markov-chain whose states correspond to the different homogeneous configurations of the population in which everyone plays the same strategy. Given the $n_{S}=25$ strategies $s_{i}={\left(t,k\right)}_{i}$, the transition matrix Γ of size $n_{S}\times n_{S}$ encodes all transition probabilities for the population to move from one homogeneous state into another. Specifically, $\Gamma_{i,j}={\left(n_{S}-1\right)}^{-1}\rho_{i,j}$ (i≠j with $\Gamma_{i,i}=1-\sum_{j}\Gamma_{i,j}$) where $\rho_{i,j}$ is probability that a homogeneous population playing $s_{i}$ will fixate into a homogeneous population playing $s_{j}$ once a mutant playing ${\;s}_{j}$ appears in that population. The results presented in Figure 3, Figure 4, Figure 5 were all obtained using the rare mutation approximation (see also the supplemental information Figures). Notwithstanding, we show in Figure S8 the excellent correspondence between the full numerical simulations^79^ and the rare-mutation approximation, which extends validity of the analytical results to mutation values well beyond what one would intuitively expect based on the principles of the approximation. For details on the analytic and numeric methods see for instance EGTtools and its documentation.^82^

#### Gradient of selection

The finite population gradient of selection G(k) is defined as the difference between the likelihood of increasing and decreasing the number k of individuals adopting the focal strategy by 1 in the population. In Figure 5 the focal strategy is (5,3): $G\left(k\right)=T^{+}\left(k\right)\;–\;T^{-}\left(k\right)\text{.}$ When G(k)>0 the fraction of (5,3) strategies is likely to increase, whereas when G(k)<0 the opposite is expected.^78^
