# Supplementary material for: Evolution of a theory of mind
Source: iScience. 2024 Jan 11;27(2):108862. doi: 10.1016/j.isci.2024.108862 (PMC10830857; doi:10.1016/j.isci.2024.108862)
Supplement: Document S1. Figures S1–S19 [file mmc1.pdf]

iScience, Volume *27*

Supplemental information

Evolution of a theory of mind

Tom Lenaerts, Marco Saponara, Jorge M. Pacheco, and Francisco C. Santos

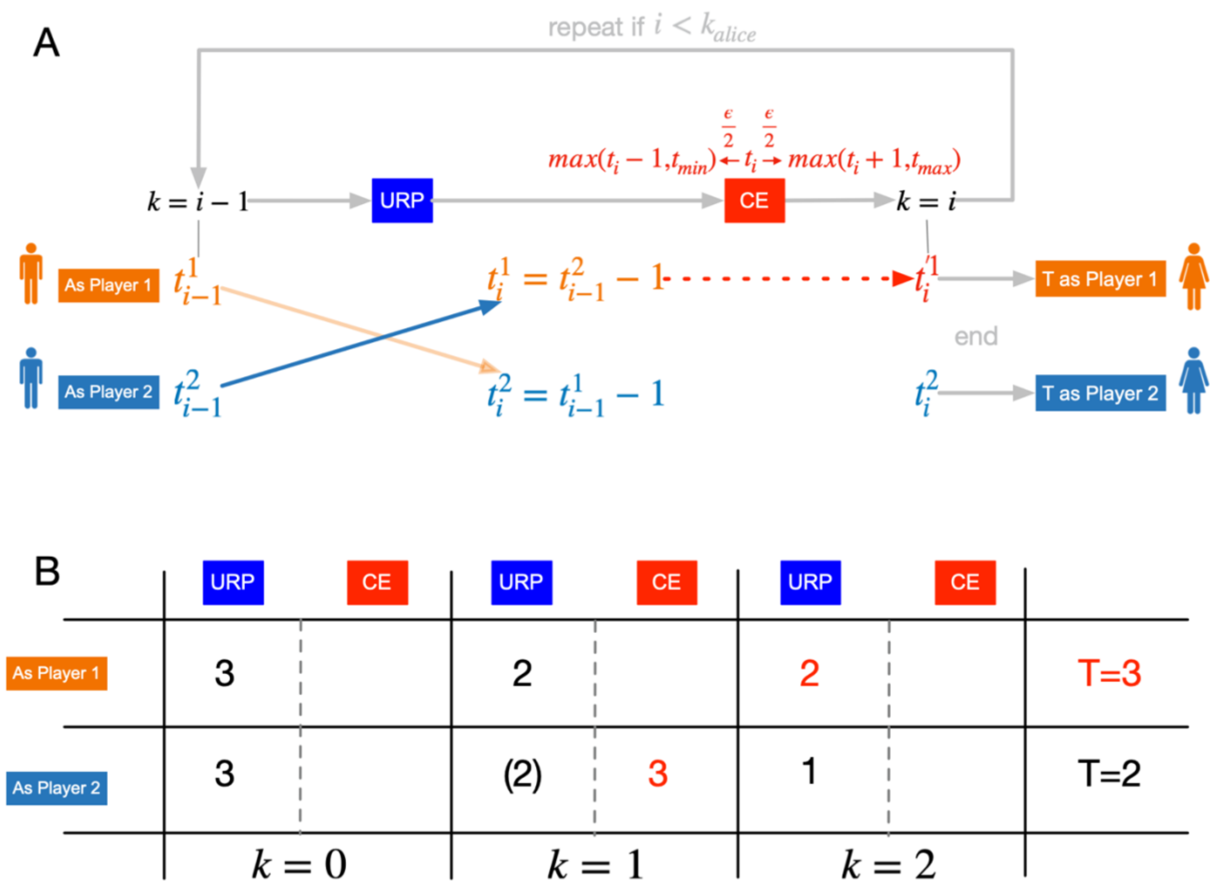

Figure S1. The unconditional reasoning process (URP), related to Figure 2. **A.** The URP kernel assumes that an individual will always try to take as early as one step before what she believes is the moment when the co-player may take. Thus, at $\boldsymbol{k = i}$, $\boldsymbol{t_i^1 = t_{i-1}^2 - 1}$ as Player 1 and $\boldsymbol{t_i^2 = t_{i-1}^1 - 1}$ as Player 2, with cognitive errors occurring with probability $\frac{\varepsilon}{2}$ as discussed in Figure 2B. **B.** Assuming Alice's strategy to be $(\boldsymbol{t}, \boldsymbol{k}) = (\mathbf{3}, \mathbf{2})$, this panel shows how the URP kernel works in a repeated manner, starting with the assumptions at $\boldsymbol{k} = \mathbf{0}$. As in Figure 2B, Alice assumes that the co-player has the same beliefs but can only reason to $\boldsymbol{k} = \mathbf{1}$ and that he has the same RP as her. So as Player 1 at $\boldsymbol{k} = \mathbf{1}$, she believes that Bob will think she will take as early as Step 3, which will make him take as early as Step 2 (blue diagonal line). The same URP is used to know what to believe as Player 2 (orange diagonal line) at $\boldsymbol{k} = \mathbf{1}$, giving in principle the same results. Yet as mentioned, a cognitive error may occur, which here happens when she infers what she believes Bob will do as Player 2 at $\boldsymbol{k} = \mathbf{1}$; She thinks he will take as early as Step 3 as opposed to Step 2. Now to decide what to do herself in both roles given her beliefs, she again applies URP arriving at taking as early as Step 2 as Player 1 (one step earlier as what she beliefs Bob would do as Player 2) and Step 1 as Player 2 (one step earlier as what she beliefs Bob would do as Player 1) at $\boldsymbol{k} = \mathbf{2}$. Taking as early as Step 2 as Player 1 in the ICG translates to taking at Step 3 as this is the moment when Alice can act as Player 1. Similarly for her action as Player 2, where she will act at Step 2 as this is the earliest moment when she can act as Player 2.

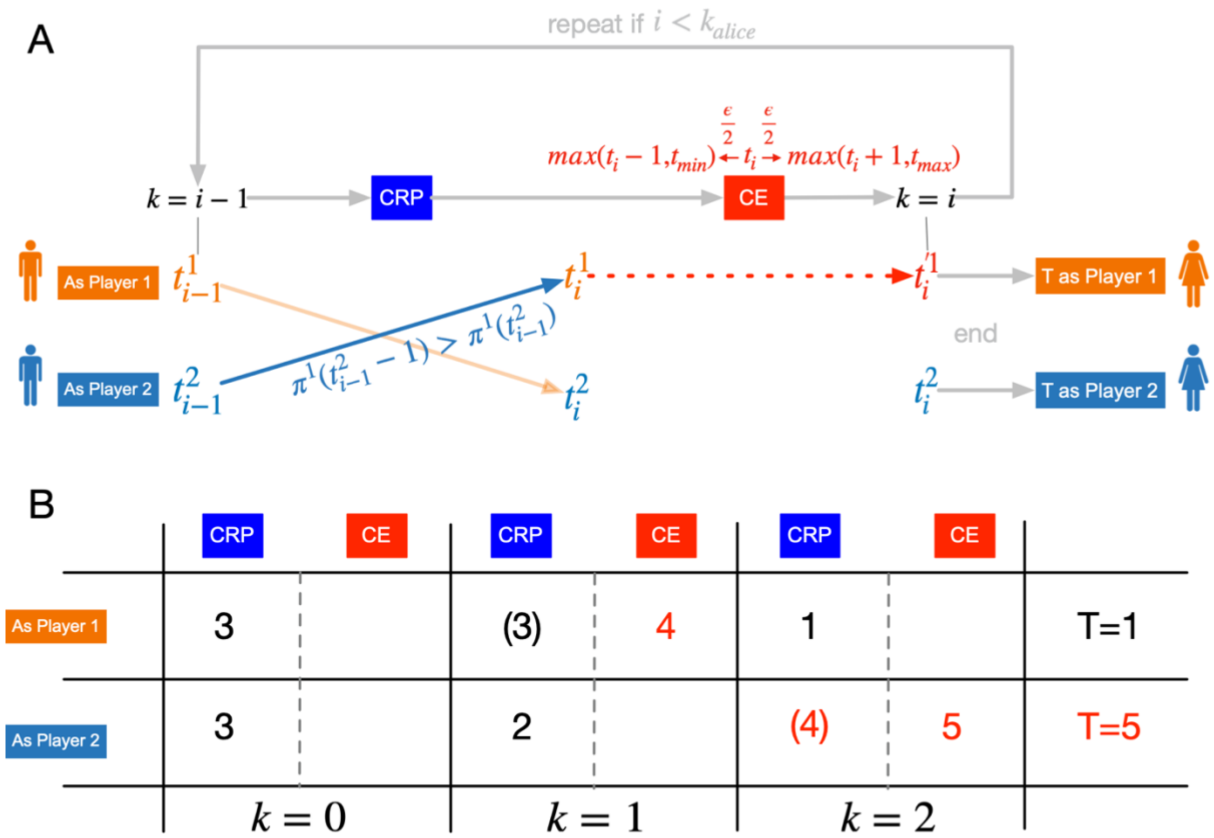

Figure S2. The payoff conditional reasoning process (CRP), related to Figure 2. A. The CRP kernel assumes that an individual will either take at the same moment as the co-player or one step before depending on which of the two gives the best payoff. So this kernel looks for a best response. Thus, at $\boldsymbol{k = i}$ as Player 1, the individual will $\boldsymbol{t^1_i = t^2_{i-1} - 1}$ when $\boldsymbol{\pi^1(t^2_{i-1} - 1)} > \boldsymbol{\pi^1(t^2_{i-1})}$ otherwise $\boldsymbol{t^1_i = t^2_{i-1}}$. Again, errors may occur with probability $\frac{\varepsilon}{2}$ as discussed in Figure 2B. **B.** Assuming Alice's strategy to be $(\boldsymbol{t, k}) = (\boldsymbol{3, 2})$, this panel shows how the CRP kernel works in a repeated manner, starting with the assumptions at $\boldsymbol{k = 0}$. Considering the Payoffs in Figure 1A, Alice reasons that as Player 1 at $\boldsymbol{k = 1}$, she gains more to take at the same time as Bob (blue diagonal line): She will obtain $\boldsymbol{\pi^1(t^2_0) = 1.6}$ as opposed to $\boldsymbol{\pi^1(t^2_0 - 1) = 0.2}$. She assumes that Bob will reason in the same way, which thus means that she believes that Bob will take as early as Step 3 at $\boldsymbol{k = 1}$ as Player 1. To know what Bob will do as Player 2, she performs the same comparison (orange diagonal line) but now using the payoffs she would obtain as Player 2: Since she believes that a co-player will take as early as Step 3 as Player 1 at $\boldsymbol{k = 0}$, she compares $\boldsymbol{\pi^2(t^1_0) = 0.4}$ and $\boldsymbol{\pi^2(t^1_0 - 1) = 0.8}$. As the latter gives her a higher payoff, she reasons that it is best to take one step before. Thus, at $\boldsymbol{k = 1}$, as Player 2, she believes that Bob will take as early as Step 2. Yet again cognitive errors may have occurred in the RP and this time it happened when she was reasoning what Bob would do as Player 1: She thinks he will act as early as Step 4 as opposed to Step 3. This cognitive error will influence the outcome of the RP at $\boldsymbol{k = 2}$, as visualized in this panel. The payoff comparison as Player 2 will lead her to stick to Step 4 like her co-player, while as Player 1 she believes she will gain more by taking one step before her co-player, which is now Step 1. Still errors may occur, and the result of reasoning leads her to believe it is best to take as early as Step 1 as Player 1 and as early as Step 5 as Player 2. Both inferences map directly to moments of playing T as she can take at Step 1 as Player 1 and at Step 5 as Player 2.

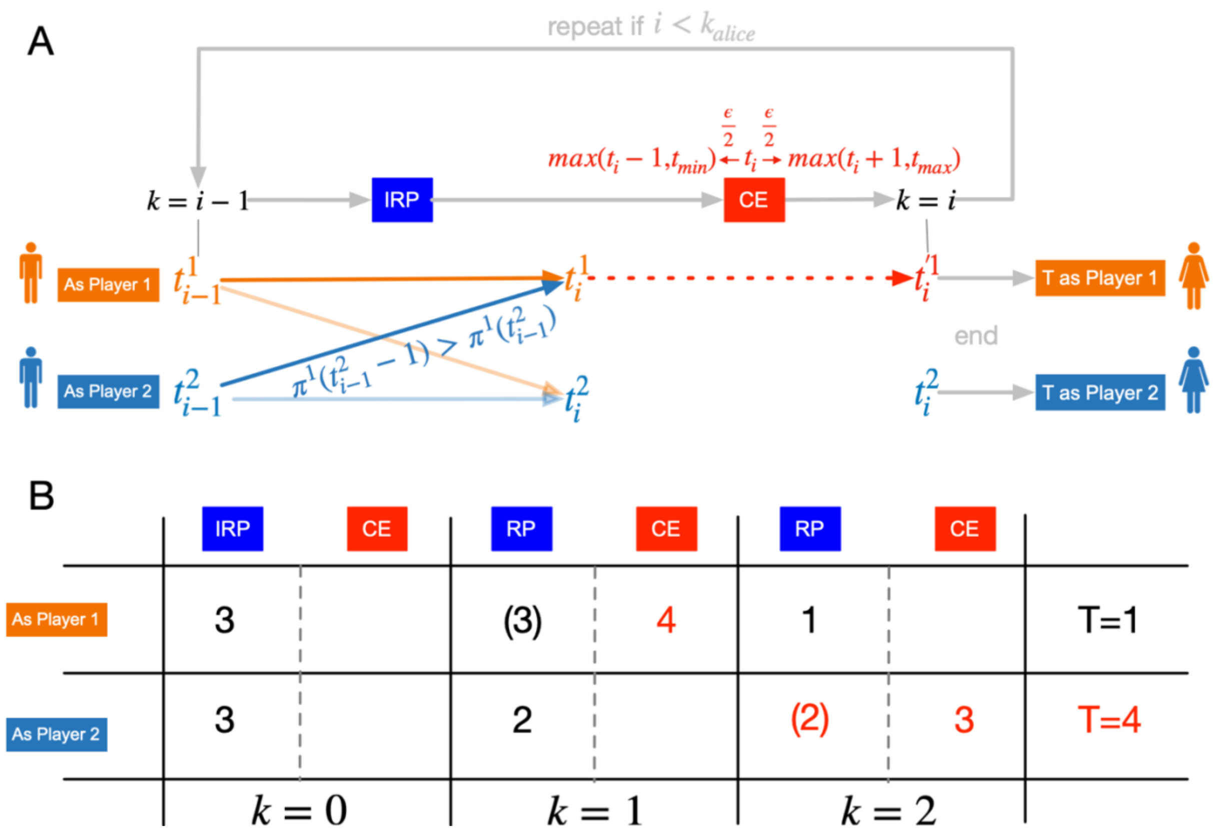

Figure S3. The inertia reasoning process (IRP), related to Figure 2. **A.** The IRP kernel will assume that an individual either takes as early as it inference at a lower reasoning level or one step before the co-player, depending on whether switching to that choice gives a payoff better than what she would get when the co-player will decide to take. Thus, at $\boldsymbol{k} = \boldsymbol{i}$ as Player 1, the individual will $\boldsymbol{t_i^1} = \boldsymbol{t_{i-1}^2} - \mathbf{1}$ when $\boldsymbol{\pi^1}(\boldsymbol{t_{i-1}^2} - \mathbf{1}) > \boldsymbol{\pi^1}(\boldsymbol{t_{i-1}^2})$ or will remain inert, i.e. $\boldsymbol{t_i^1} = \boldsymbol{t_{i-1}^1}$. The inferences as Player 2 are the same and errors may occur with probability $\frac{\varepsilon}{2}$ as discussed in Figure 2B. **B.** Assuming Alice's strategy to be $(\boldsymbol{t}, \boldsymbol{k}) = (\mathbf{3}, \mathbf{2})$, this panel shows how the IRP kernel works in a repeated manner, starting with the assumptions at $\boldsymbol{k} = \mathbf{0}$. Alice compares, in her role as Player 1 at $\boldsymbol{k} = \mathbf{1}$, $\boldsymbol{\pi^1}(\boldsymbol{t_0^2}) = \mathbf{1.6}$ with $\boldsymbol{\pi^1}(\boldsymbol{t_0^2} - \mathbf{1}) = \mathbf{0.2}$. As she does not see an improvement by trying to take earlier as Player 2, she believes that she should continue to act as early as what she would have done at $\boldsymbol{k} = \mathbf{0}$, i.e. $\boldsymbol{t_1^1} = \boldsymbol{t_0^1} = \mathbf{3}$. She believes that Bob will reason in the same was and thus take as early as Step 3 at $\boldsymbol{k} = \mathbf{1}$ as Player 1. Yet, a cognitive error occurs when reasoning as Player 1 (in red): She thinks he will act as early as Step 4 as opposed to Step 3. As Player 2 at $\boldsymbol{k} = \mathbf{1}$, she compares $\boldsymbol{\pi^2}(\boldsymbol{t_0^1}) = \mathbf{0.4}$ and $\boldsymbol{\pi^2}(\boldsymbol{t_0^1} - \mathbf{1}) = \mathbf{0.8}$, where the latter provides a higher payoff. So she sees a benefit to take before the co-player and she reasons that Bob will see the same and decide to take one step before her. Thus, as Player 2, Bob will take as early as Step 2 (and no cognitive error occurs). Now, at $\boldsymbol{k} = \mathbf{2}$, reasoning as Player 1, she sees that $\boldsymbol{\pi^1}(\boldsymbol{t_1^2} - \mathbf{1}) = \mathbf{0.4}$ is better than $\boldsymbol{\pi^1}(\boldsymbol{t_1^2}) = \mathbf{0.2}$, leading to the decision to take as early as Step 1. Reasoning as Player 2, based on her belief about Bob, she observes that $\boldsymbol{\pi^2}(\boldsymbol{t_1^1} - \mathbf{1}) = \mathbf{0.4}$ is worse than $\boldsymbol{\pi^2}(\boldsymbol{t_1^1}) = \mathbf{3.2}$, leading to the decision to act in the same way as what she inferred at $\boldsymbol{k} = \mathbf{1}$ in the role of Player 2, i.e. $\boldsymbol{t_2^2} = \boldsymbol{t_1^2} = \mathbf{2}$. Still errors may occur, and the result of reasoning leads her to believe it is best to take as early as Step 1 as Player 1 and as early as Step 3 as Player 2. While the inference as Player 1 maps directly to her moment of playing T, as Player 2 given the inference result, she will actually take at Step 4.

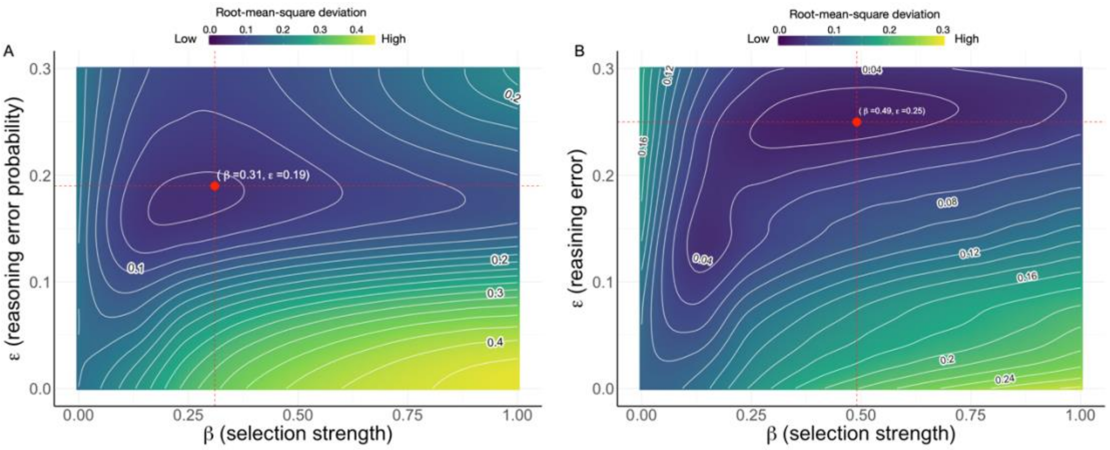

Figure S4. Root-mean-square deviation between experimental results [1] and the present evolutionary ToM model, related to Figure 3. **A.** Results for the ICG with $\boldsymbol{L} = \mathbf{4}$. The red dot matches the best root-mean-square deviation results, corresponding to the selection strength $\boldsymbol{\beta} \simeq \mathbf{0.31}$ and reasoning error $\boldsymbol{\varepsilon} \simeq \mathbf{0.19}$. **B.** Results for the ICG with $\boldsymbol{L} = \mathbf{6}$. The red dot matches the best root-mean-square deviation results, corresponding to the selection strength $\boldsymbol{\beta} \simeq \mathbf{0.49}$ and reasoning error $\boldsymbol{\varepsilon} \simeq \mathbf{0.25}$.

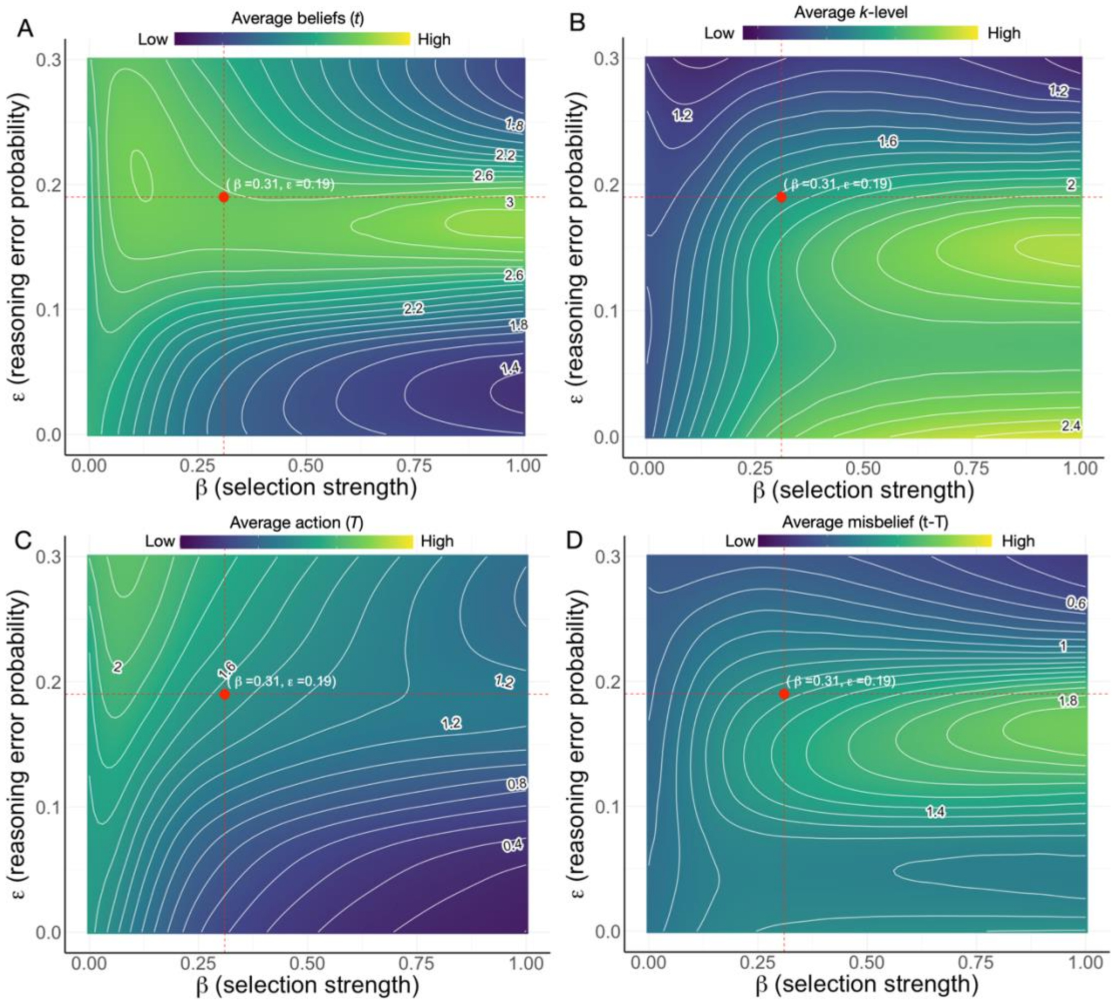

Figure S5 Average belief t, k-levels, action T and misbeliefs $(t - T)$ in the ICG $(L = 4)$ employed in the main text for a wide range of (β, ε) parameter values, related to Figure 3. **A.** The average Belief t. **B.** The average k-level. **C.** The average action T. **D.** The average misbelief, i.e. the difference between the average belief and average action. In each panel, the experimentally calibrated combination $(\beta = 0.31, \varepsilon = 0.19)$ inferred from McKelvey and Palfrey [1] is highlighted by a red circle. $Z = 500$ in all panels. Note that in this figure, beliefs and actions are within the set $\{0,1,2,3,4\}$.

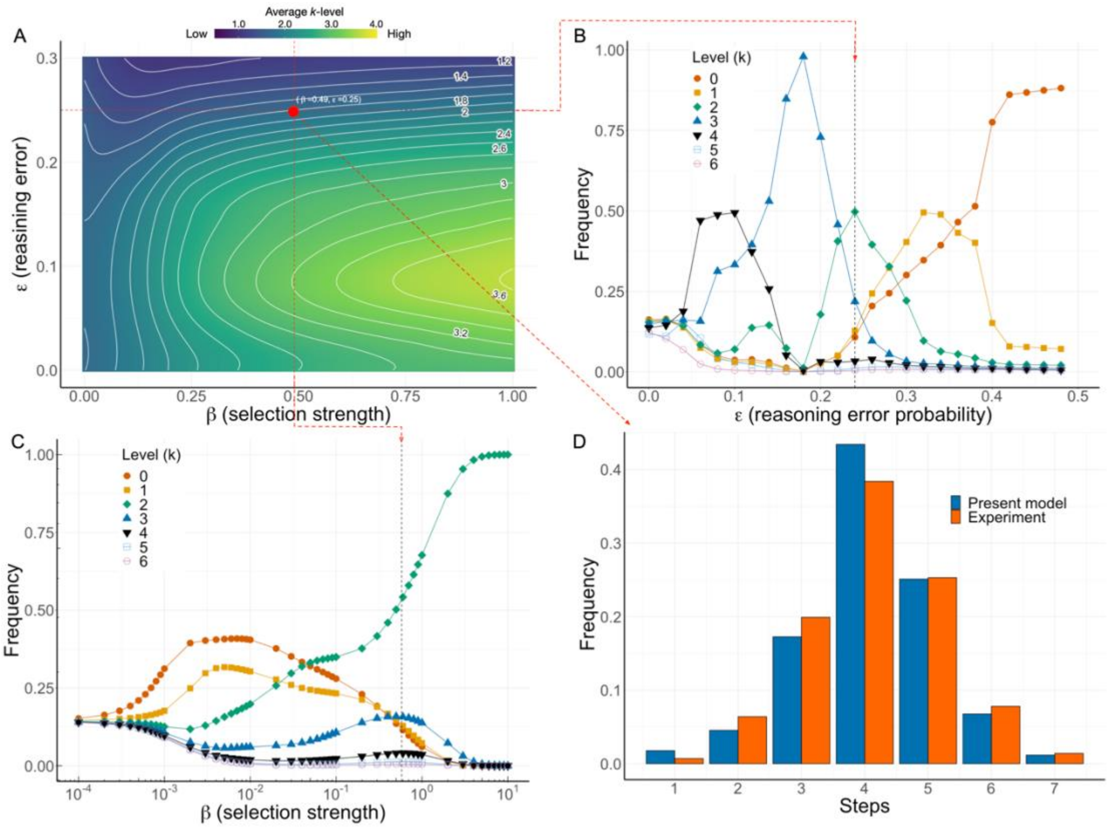

Figure S6. Evolution of ToM in ICG with $L = 6$, related to Figure 3. **A.** Average k-level emerging from evolving a population of $Z = 500$ individuals as a function of the selection pressure β and the cognition error probability ε. Fitting our stationary distribution of Steps to those deduced from [1] leads to the comparative plot in Panel **D**, with optimum values ($\beta^* \approx 0.49, \varepsilon^* \approx 0.25$) depicted with a red circle in Panel **A.** Panels **B** and **C** portray the k-level distributions as a function of ε and β, respectively, in each case keeping the other parameter at the experimentally calibrated value.

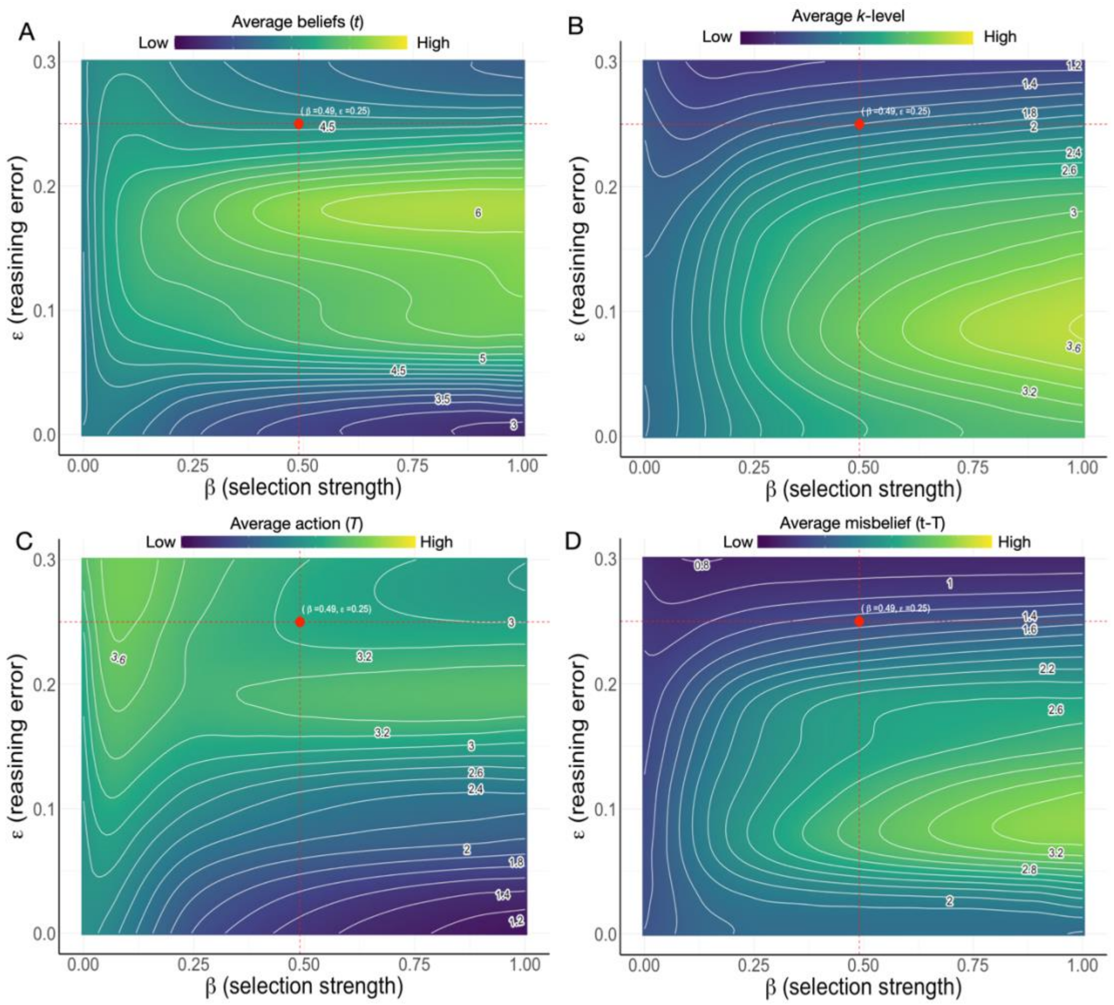

Figure S7. Same quantities as in Figure S5 are plotted here for the ICG with $L = 6$ for a wide range of (β, ε) settings, related to Figure 3. We also use the same notation as in Figure S5, except that here, in each panel, the red circle indicates the experimentally calibrated combination ($\beta = 0.49, \varepsilon = 0.25$) inferred from McKelvey and Palfrey [1]. $Z = 500$ in all panels. Note that in this figure, beliefs and actions are within the set $\{0,1,2,3,4,5,6\}$.

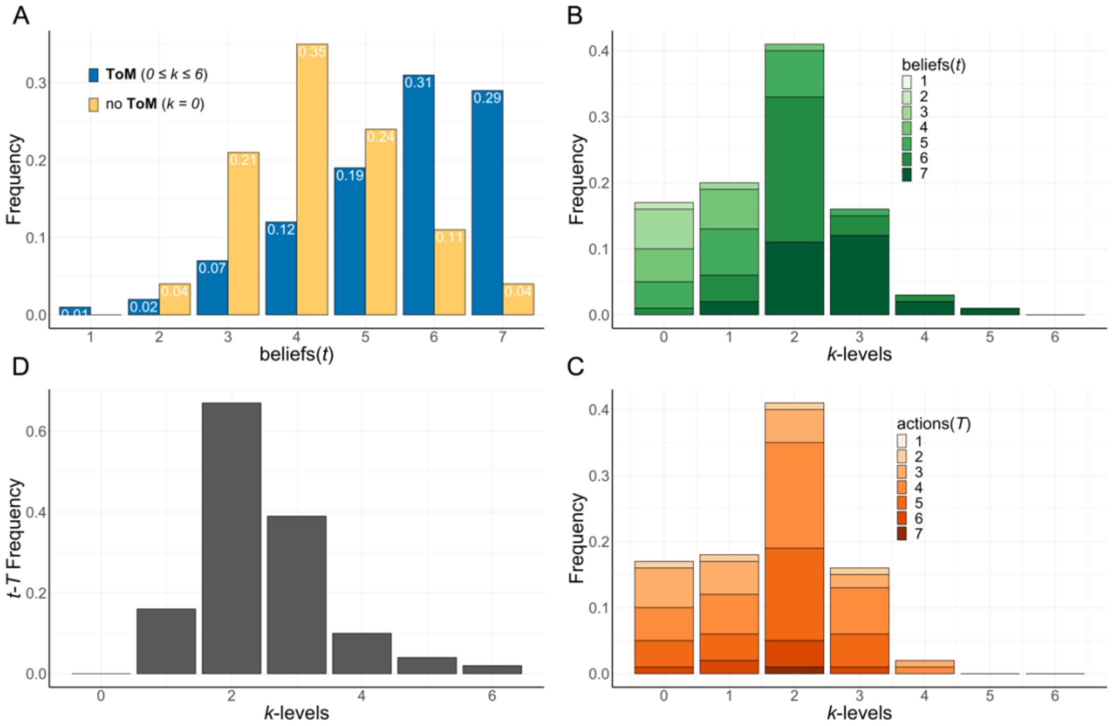

Figure S8. Evolution of strategies with a ToM in the ICG with $L = 6$, related to Figure 4. **A.** Direct comparison between the evolution of the beliefs in the presence $(0 \leq k \leq 6)$ and absence $(k = 0)$ of a ToM. Opening the possibility for strategies encompassing a ToM leads to very different distributions of beliefs, similar to what was obtained for the case $L = 4$ depicted in Figure 4. This figure uses the same notation and conventions as those used in Figure 4.

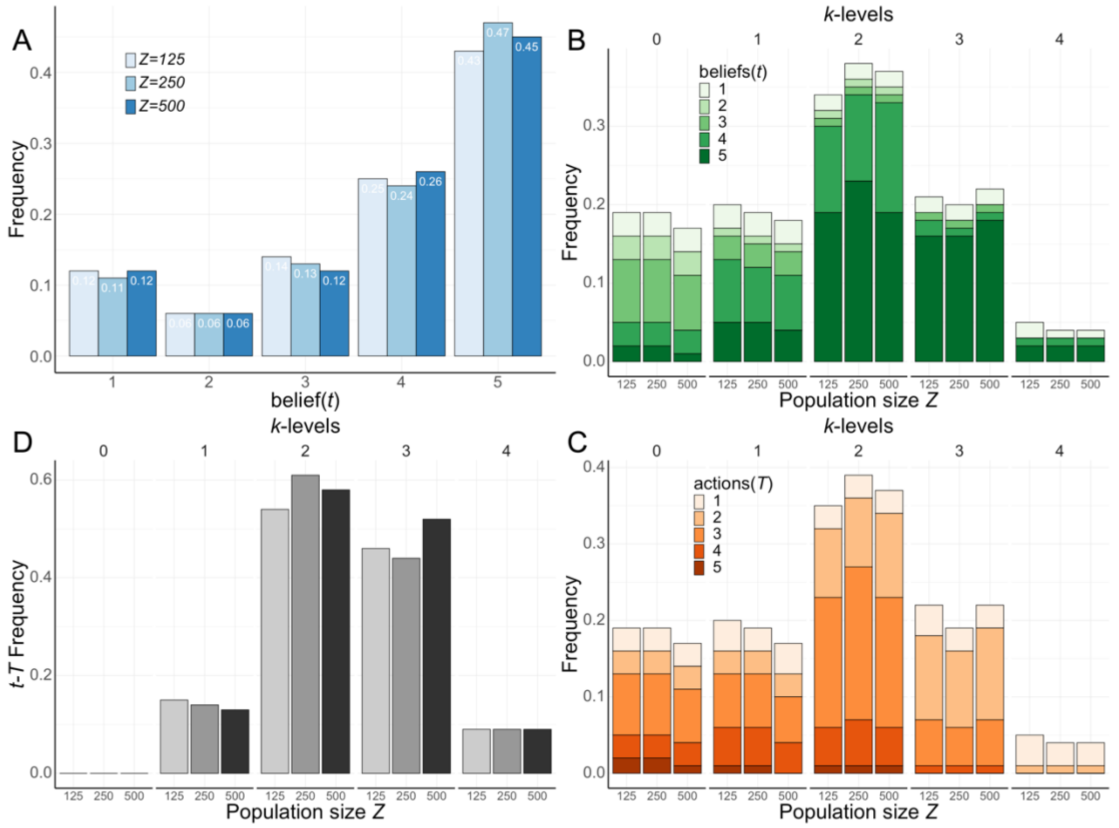

Figure S9. Population size effects on the ICG game, related to Figure 4. We vary here the population size for the $\boldsymbol{L} = \mathbf{4}$ ICG game, i.e., $\boldsymbol{Z} \in \{\mathbf{125}, \mathbf{250}, \mathbf{500}\}$. We use the same notation as in Figure 4. As can be observed, all results remain equivalent for the different population sizes, although the optimal $(\boldsymbol{\beta}, \boldsymbol{\varepsilon})$-combination inferred from the behavioural experiment data is affected. With increasing population sizes, the corresponding optimal selection strength values become respectively $\boldsymbol{\beta}^*_{125} = \mathbf{0.98}$, $\boldsymbol{\beta}^*_{250} = \mathbf{0.52}$ and $\boldsymbol{\beta}^*_{500} = \mathbf{0.31}$, while $\boldsymbol{\varepsilon}$ remains almost constant ($\boldsymbol{\varepsilon}^* \approx \mathbf{0.19}$) in all three cases. Consequently, all three results were produced with $\boldsymbol{\varepsilon} = \mathbf{0.19}$.

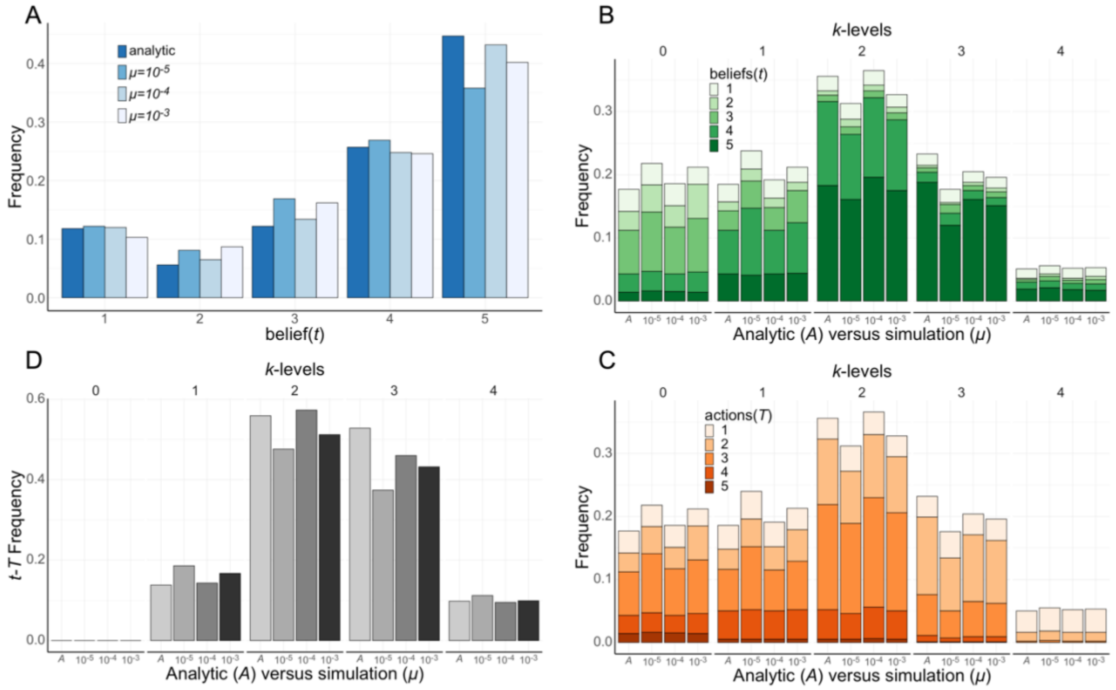

Figure S10. Numerical simulations of the model and comparison to analytical results, related to Figure 4. Panels **A** to **D** show the same as in Figure 4 yet comparing the analytical results ($\boldsymbol{A}$) for ICG with $\boldsymbol{L} = \mathbf{4}$ with the full numerical simulation results (see Methods). In the numerical simulations we used mutation probability values of $\boldsymbol{\mu} \in \{\mathbf{10^{-5}}, \mathbf{10^{-4}}, \mathbf{10^{-3}}\}$ with $\boldsymbol{Z} = \mathbf{500}$. A total of $\mathbf{1000}$ simulations with $\mathbf{10^{8}}$ iterations each were performed to calculate the stationary distributions for each setting, producing the data for the four panels. Comparison of numerical simulations with the analytical approximation used in the main text shows that the validity of the analytical approximation extends to regimes well-beyond those one would expect from the nature of the approximation.

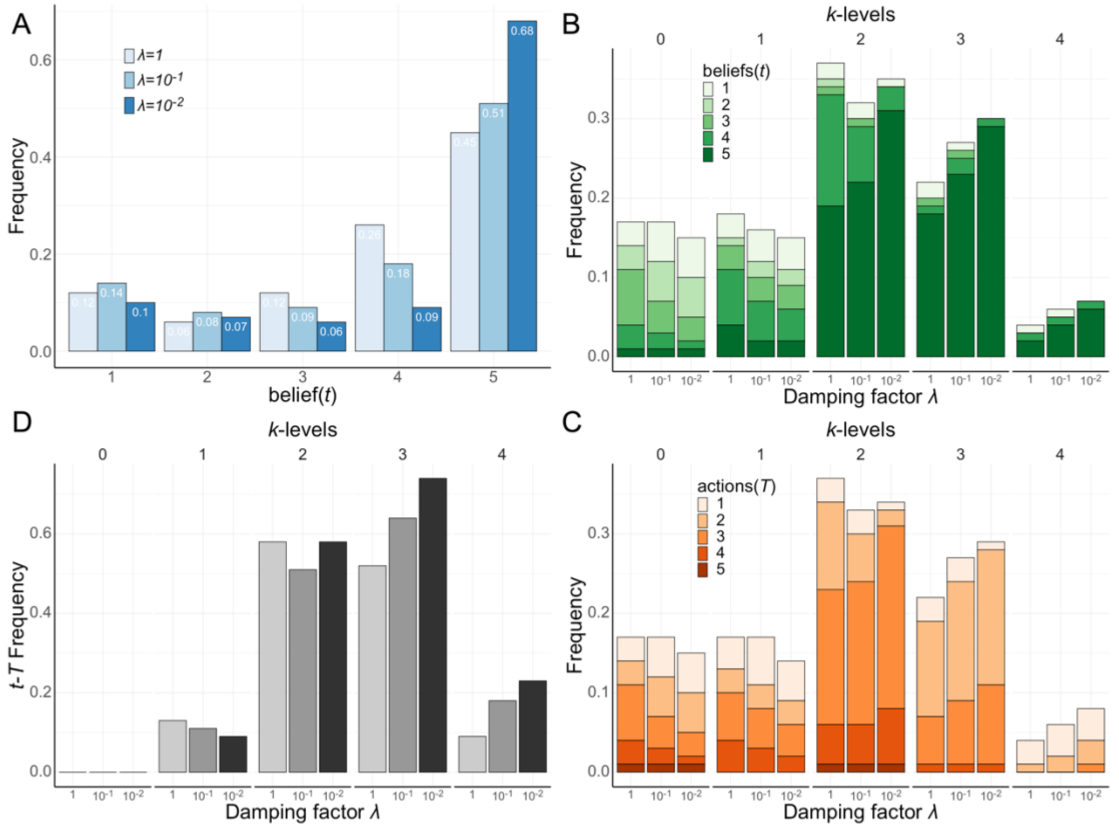

Figure S11. Effect of inhibiting transitions between $\boldsymbol{k}$-levels with respect to transitions between beliefs, related to Figure 4. Panels **A** to **D** show the same data as in Figure 4 when the transition probabilities between different strategies comprising different beliefs remain unchanged, while the corresponding transitions between strategies comprising different $\boldsymbol{k}$-levels of cognition are inhibited by a damping factor $\boldsymbol{\lambda}$. This way we qualitatively mimic the idea that changing beliefs is easier than changing one's own cognitive level. We used the values $\boldsymbol{\lambda} = \{\mathbf{1}, \mathbf{10^{-1}}, \mathbf{10^{-2}}\}$ to scale the aforementioned transitions between monomorphic configurations in the framework of the analytical framework employed in the main text (see Material and Methods). The results show that the overall scenario remains qualitatively unchanged as we inhibit the transition up to 2 orders of magnitude. In all panels, the population size is $\boldsymbol{Z} = \mathbf{500}$, $\boldsymbol{\beta}^* = \mathbf{0.31}$ and $\boldsymbol{\varepsilon}^* = \mathbf{0.19}$.

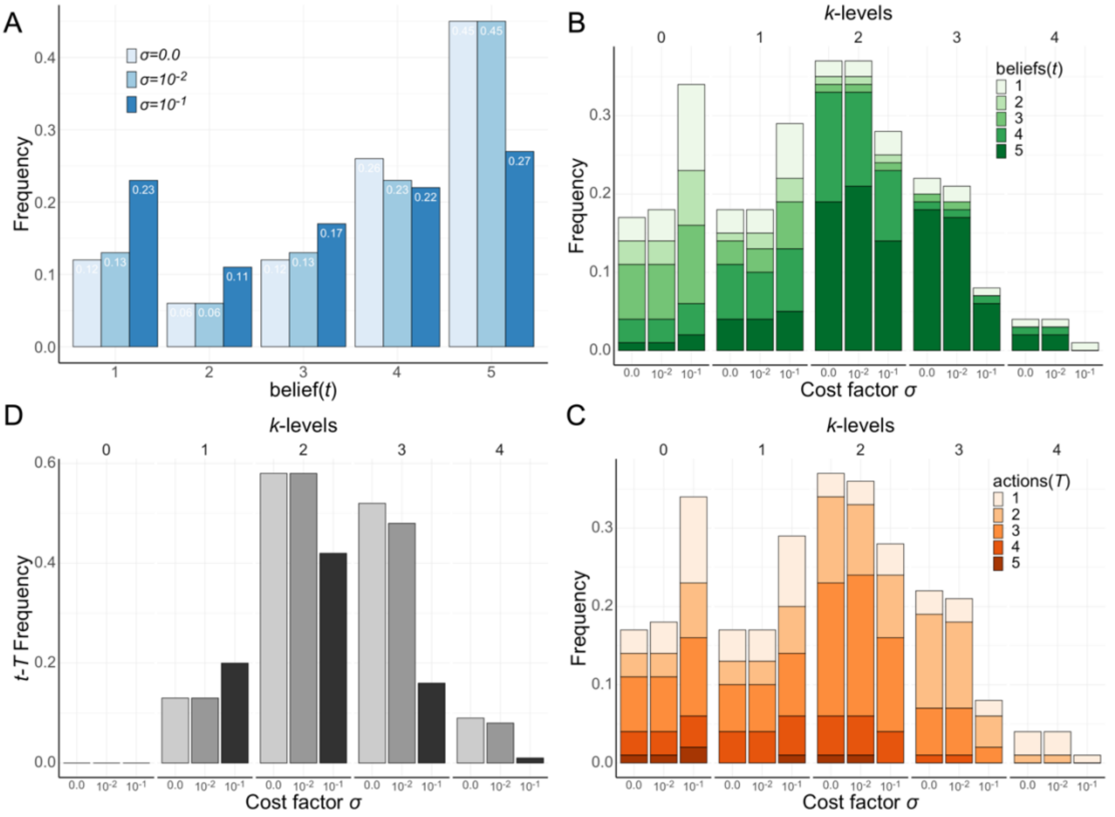

Figure S12. The effect of reasoning costs on the ICG game, related to Figure 4. We introduce a linear reasoning cost $\boldsymbol{c} = \boldsymbol{\sigma}\,\boldsymbol{k}$ and compare the results for the $\boldsymbol{L} = \mathbf{4}$ ICG game with the no cost model dynamics employed in the main text. Results show the comparison for $\boldsymbol{\sigma} = \{\mathbf{0}, \mathbf{10^{-2}}, \mathbf{10^{-1}}\}$. We use the same notation as in Figure 4. With increasing $\boldsymbol{\sigma}$, lower $\boldsymbol{k}$-levels become increasingly favoured, while beliefs approach a more uniform distribution where optimism bias is mostly absent. The plots for each value of $\boldsymbol{\sigma}$ were obtained for $\boldsymbol{\beta}^* = \mathbf{0.31}$ and $\boldsymbol{\varepsilon}^* = \mathbf{0.19}$ for all values of $\boldsymbol{\sigma}$. In all panels, $\boldsymbol{Z} = \mathbf{500}$.

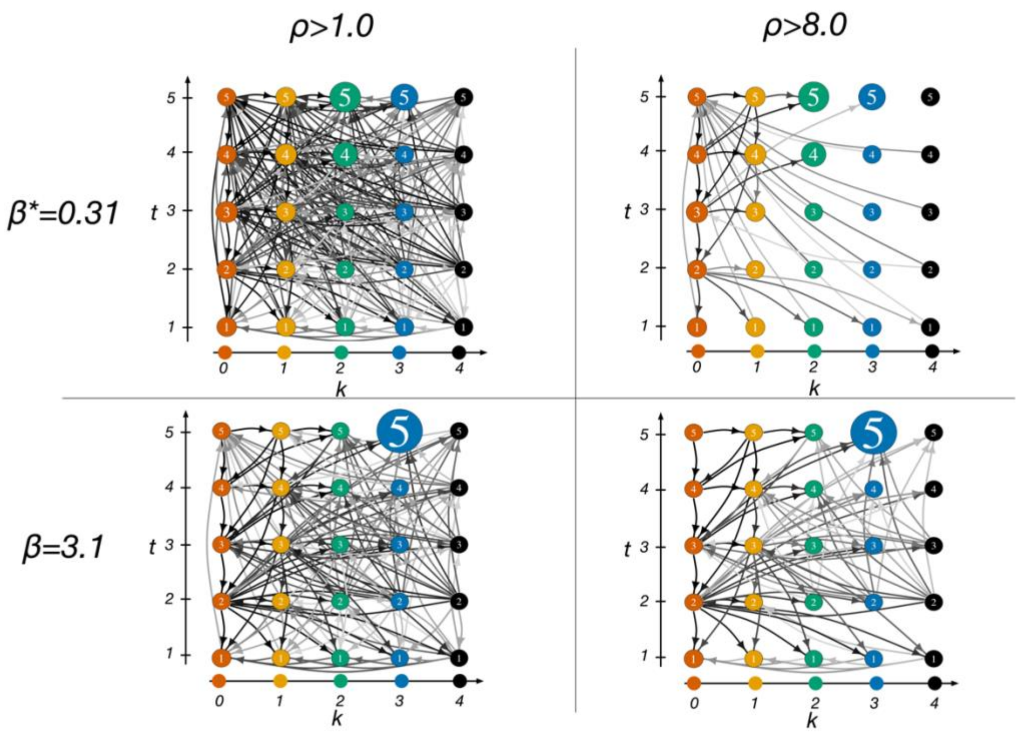

Figure S13. Invasion graphs involving all (25) strategies $(\boldsymbol{t}, \boldsymbol{k})$ in the ICG with $\boldsymbol{L} = \mathbf{4},$ related to Figure 5. The number in each node refers to the value of $\boldsymbol{t}$ and the color the value of $\boldsymbol{k}.$ The Top panels were obtained for the optimum value of the selection pressure ($\boldsymbol{\beta} = \mathbf{0.31}$) whereas the Bottom panels show results for high selection pressure ($\boldsymbol{\beta} = \mathbf{3.1}$). The size of each node is proportional to its prevalence in the stationary distribution (see STAR Methods). Arrows representing transitions between strategies are displayed with a gray-shaded gradient, where darker arrows represent stronger transitions. In the Left column, only transition probabilities with a strength larger than neutral (that is, larger than 1/Z) are shown. The invasion graphs in Right column zoom in on the transition probabilities with a strength 8 times larger than neutral. What can be seen in the Top Right graph is that the larger transition probabilities are going from i) low $\boldsymbol{k}$ and high $\boldsymbol{t}$ to low $\boldsymbol{k}$ and low $\boldsymbol{t}$, ii) from high $\boldsymbol{k}$ and low $\boldsymbol{t}$ to the first and iii) from low $\boldsymbol{k}$ and high $\boldsymbol{t}$ to intermediate $\boldsymbol{k}$-levels (up until $\boldsymbol{k} = \mathbf{2}$) with high $\boldsymbol{t}$. Essentially i and ii show a kind of cyclic behaviour and iii provides a flux outward towards the $(\mathbf{4}, \mathbf{2})$, $(\mathbf{5}, \mathbf{2})$ and $(\mathbf{5}, \mathbf{3})$ strategies. As a result, nodes with $\boldsymbol{t} = \mathbf{4}$ and $\boldsymbol{t} = \mathbf{5}$ prevail, as well as nodes with $\boldsymbol{k} = \mathbf{2}$ and $\boldsymbol{k} = \mathbf{3}.$ Increasing $\boldsymbol{\beta}$ by one order of magnitude leads to the results shown in the lower panels, where the strategy $(\mathbf{5}, \mathbf{3})$ emerges as *Evolutionary Robust* [2,3], meaning that the outgoing edges from this node, if any, have a transition probability less than neutral (this result was numerically confirmed). At high selection pressure, the co-evolution of beliefs $(\boldsymbol{t})$ and ToM $(\boldsymbol{k})$ keeps high values of beliefs stable (unlike what would happen in the absence of a ToM) enforcing $\boldsymbol{t} = \mathbf{5}$, leading to a unique evolutionary robust correlated equilibrium $(\mathbf{5}, \mathbf{3})$. In all panels, $\boldsymbol{\varepsilon}^* = \mathbf{0.19}$ and $\boldsymbol{Z} = \mathbf{500}$.

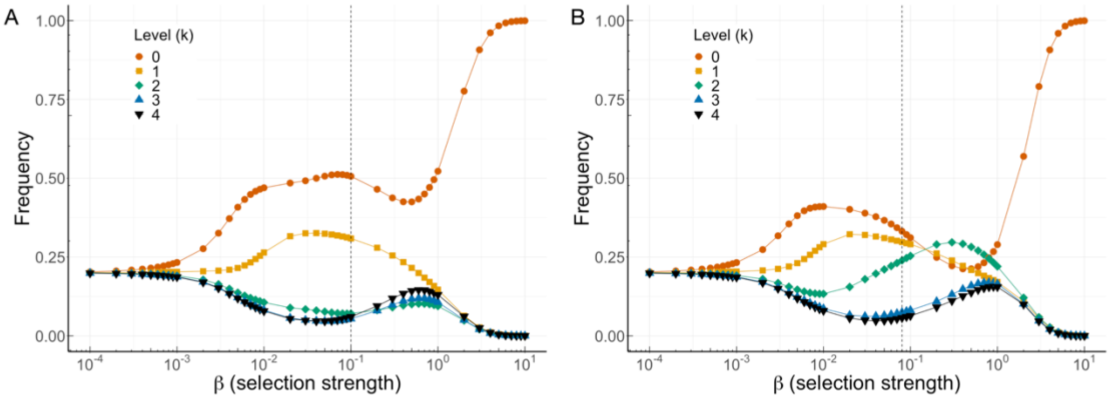

Figure S14. $\boldsymbol{k}$-level stationary distributions for different RP, related to Figure 5. This Figure shows the same information as Figure **3C** in the main manuscript but now for the unconditional RP (see Figure S1) in panel **A** and the payoff-conditional RP (See Figure S2) in panel **B**. In both panels, the dotted vertical line indicates the optimal $\boldsymbol{\beta}$-fitting for the behavioural data, i.e $\boldsymbol{\beta} = \mathbf{0.1}$ and $\boldsymbol{\beta} = \mathbf{0.08}$. The corresponding cognitive error probabilities are respectively $\boldsymbol{\varepsilon} = \mathbf{0.42}$ and $\boldsymbol{\varepsilon} = \mathbf{0.16}$. While one can observe that at the optimal fitting, there is a heterogeneity in $\boldsymbol{k}$-levels, the distribution is now favouring $\boldsymbol{k} = \mathbf{0}$ and $\boldsymbol{k} = \mathbf{1}$ as opposed to the higher reasoning levels observed for the inertia RP in Figure **3**. Additionally, higher selection strengths will shift the distribution towards $\boldsymbol{k} = \mathbf{0}$, which is associated with the (1,0) strategy.

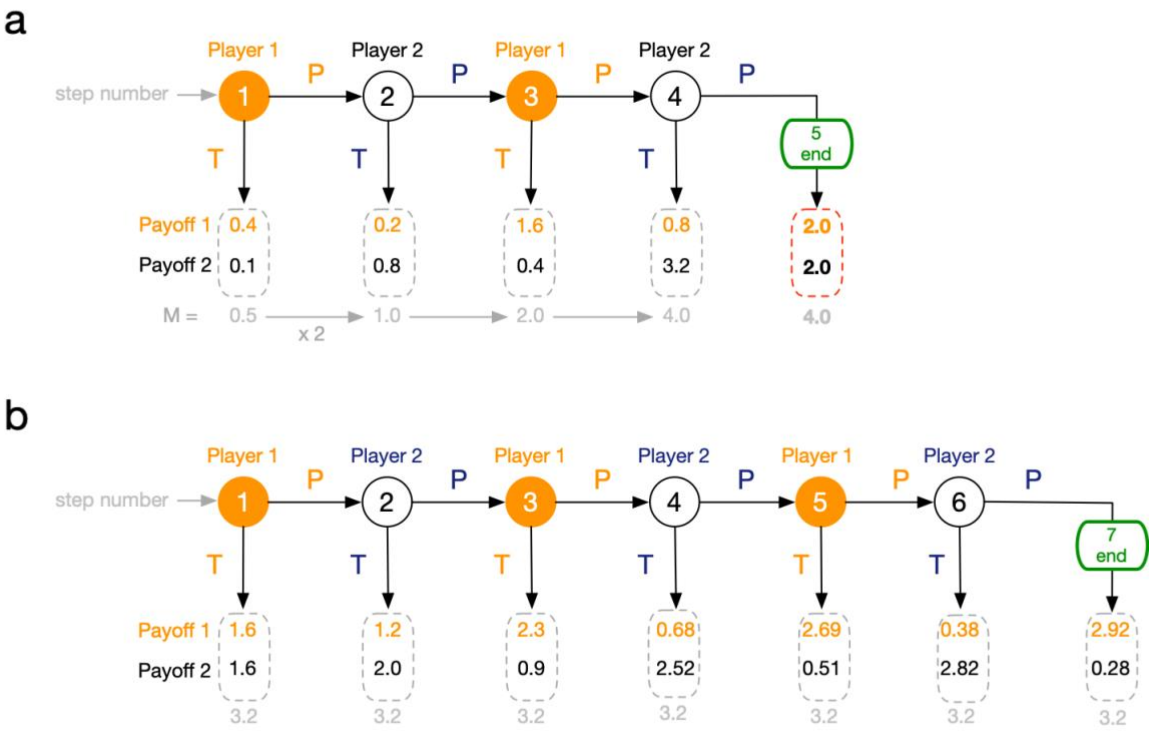

Figure S15. Variations on the centipede game, related to Figure 1. **A.** The ICG with equal split at the end. This game is the same as shown and discussed in Extended Data Figure **1** but for the end round: The last pass does not result in an 80% gain for Player 1 but in an equal division between both players. This variant of the ICG introduces the additional complexity of deciding whether they prefer a fair outcome or not. In addition, the resource does not grow in this last step. **B.** The constant-pie centipede game (CCG). In this version of the centipede game the joint resource remains the same over all steps ($M = 3.2$). Playing P in the first step results in an increasingly asymmetric division favouring the focal player.

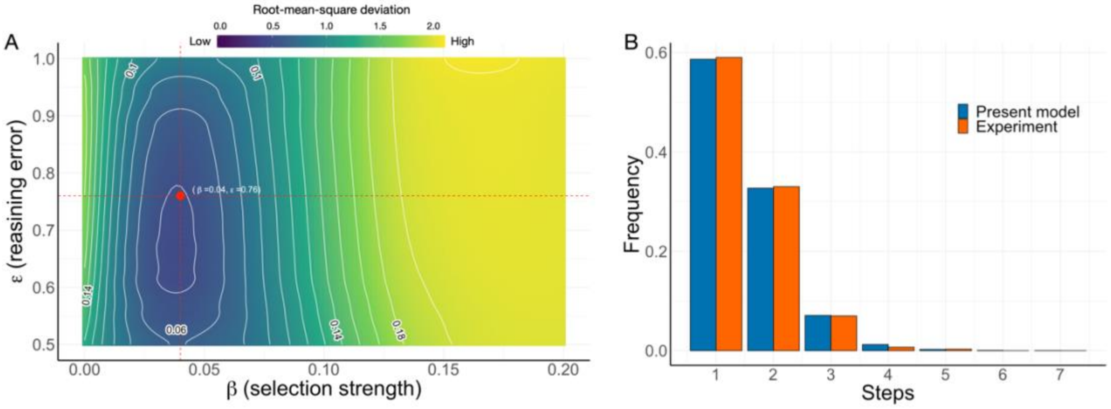

Figure S16. Calibration of the parameters associated with the CCG for $L = 6$, related to Figure 3. **A.** Root-mean-square deviation between experimental results [4] and the evolutionary ToM model for the **CCG** with $L = 6$. The red dot in panel **A** matches the best root-mean-square deviation results, corresponding to the selection strength $\beta^* \simeq 0.04$ and $\varepsilon^* \simeq 0.76$. Cognitive errors provide thus the main explanation for the fitting between the model and the experimental results.

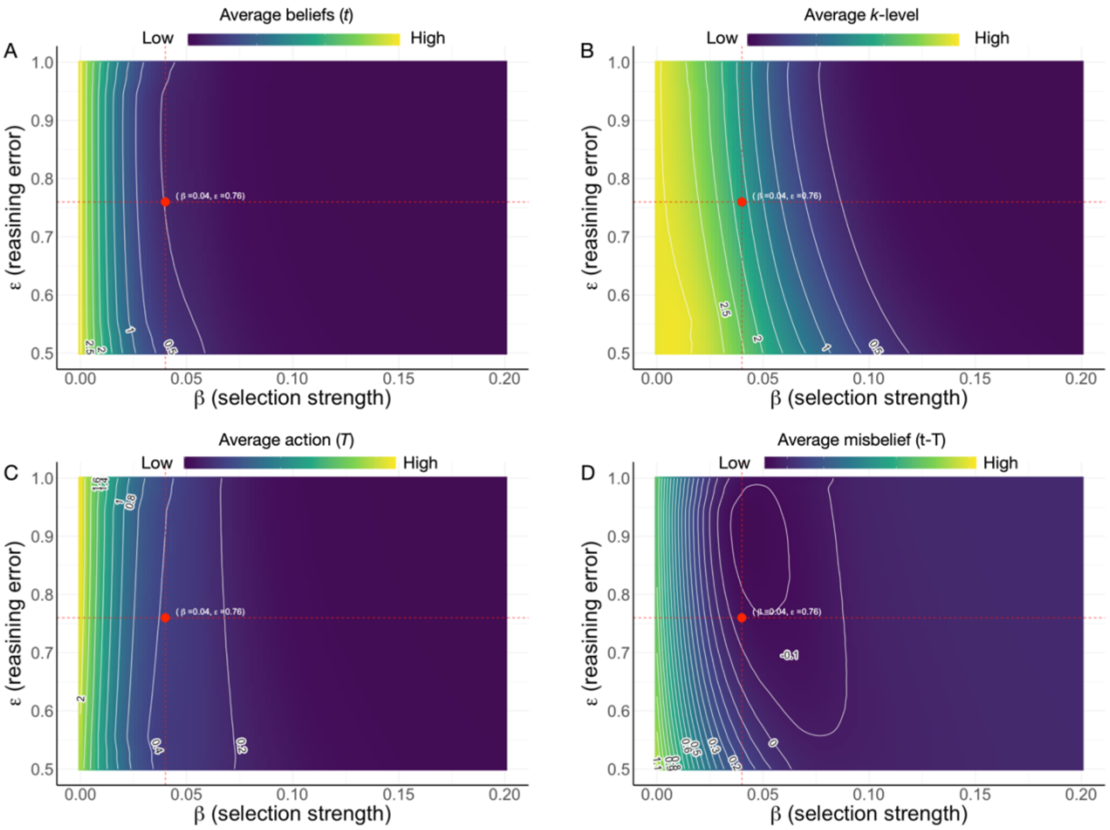

Figure S17 Same quantities as in Figures S5 and S7 are plotted here for the **CCG** with $L = 6$ for a wide range of (β, ε) values, related to Figure 3. We also use the same notation as in Figures S5 and S7. In each panel, the experimentally calibrated combination $(\beta^* = 0.04, \varepsilon^* = 0.76)$ inferred from Fey et al. [4] is highlighted by a red circle (at the top of the dashed lines). $Z = 500$ in all panels. As can be observed, the optimal fitting corresponds to beliefs that everyone will take early (Panel **A**), and extensive cognitive errors are needed in the reasoning (Panel **B**) to fit the distribution observed in Figure S16B. Overall, the decision to take is early (Panel **C**) and misbeliefs are close to zero (and can be also pessimistic, as shown in Panel **D**). Note that in this figure, beliefs and actions are within the set $\{0,1,2,3,4,5,6\}$.

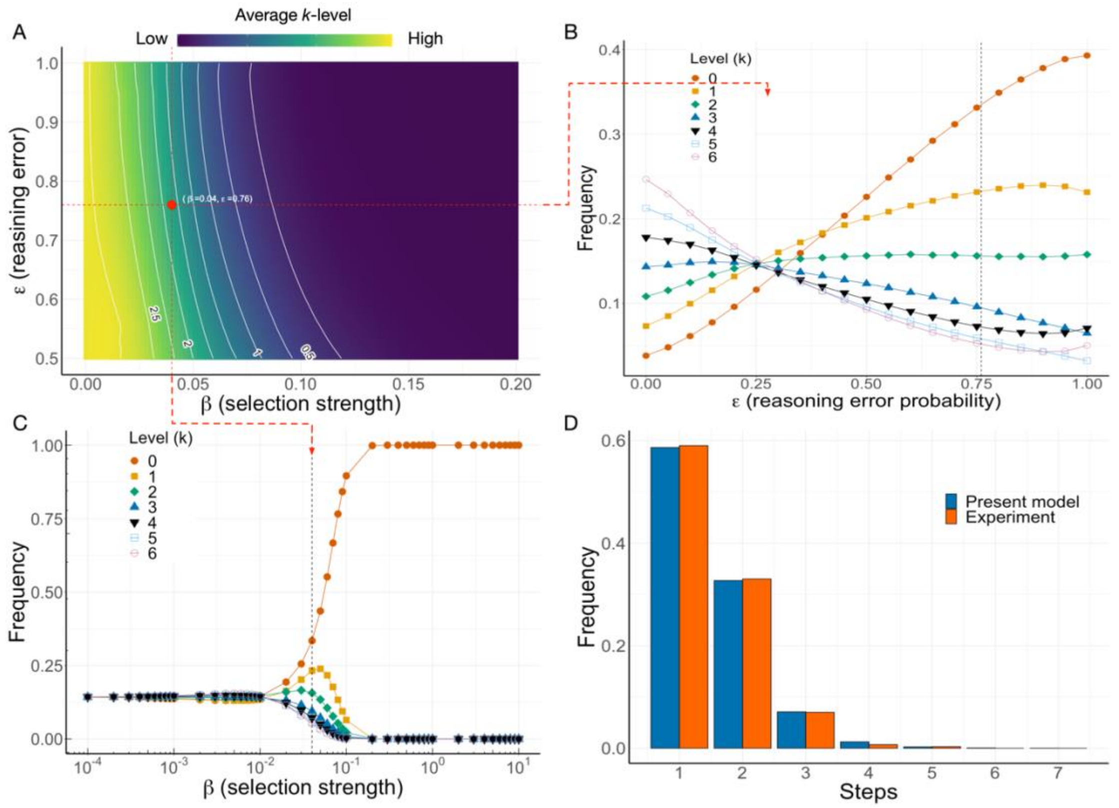

Figure S18. Evolution of a ToM in CCG, related to Figure 3. **A.** Average k-level emerging from evolving a population of $Z = 500$ individuals as a function of the selection pressure β and the cognition error probability ε. Fitting our stationary distribution of Steps to those deduced from Fey et al. [4] leads to the optimum values ($\beta^* \approx 0.04, \varepsilon^* \approx 0.74$) depicted with a red circle in panel **A**. Comparison between Fey et al. [4] and the theoretical best fit is made in panel **D**. Panels **B** and **C** portray the k-level distributions as a function of ε and β, respectively, in each case keeping the other parameter at the optimum value (β^* and ε^*, respectively).

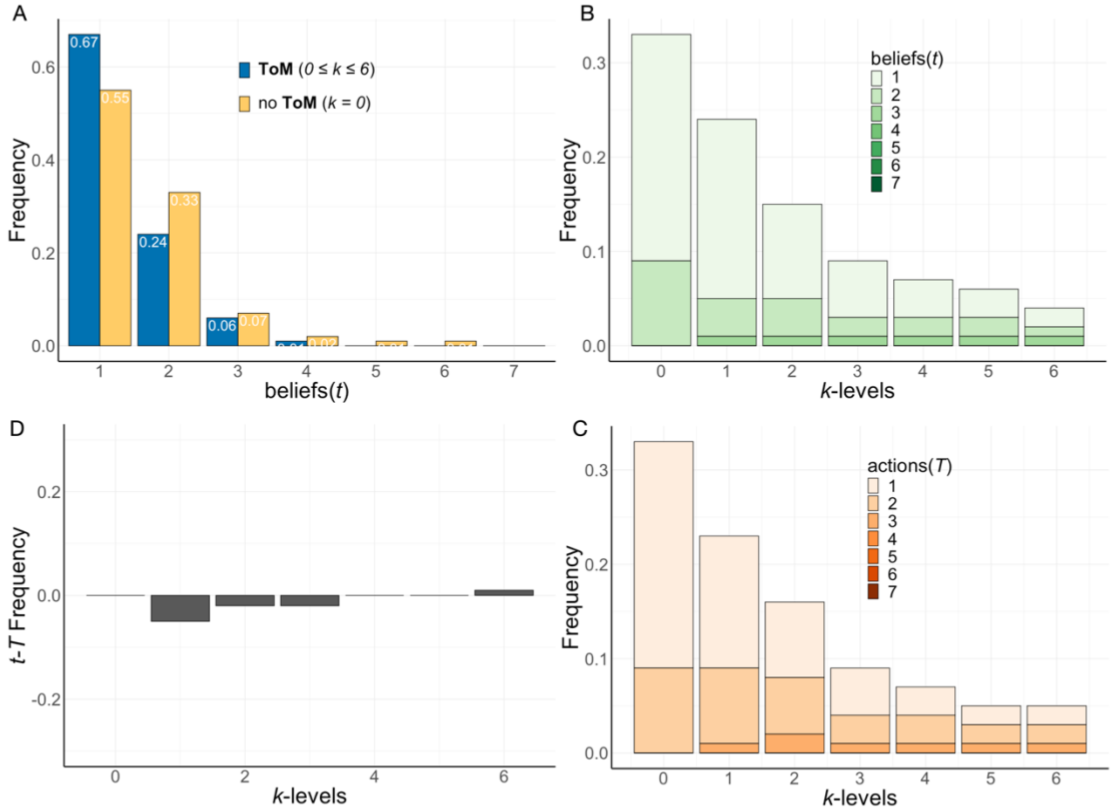

Figure S19. Evolution of strategies with a ToM in the CCG with $L = 6$, related to Figure 4. **A.** Direct comparison between the evolution of the beliefs in the absence ($k = 0$) and presence ($0 \leq k \leq 6$) of a ToM. Strategies incorporating a no ToM ($k = 0$) and ToM ($k > 0$) have similar belief distributions. **B.** Composition of the population as a function of k-level; for each k-level, the distribution of beliefs (t) is shown. Individuals adopting strategies with no ToM ($k = 0$) dominate in the population, although decreasing fractions of k-levels coexist. Beliefs in each k-level are oriented to $t = 1$ and $t = 2$. **C.** Same as **B** except that now, for each k-level, the distribution of actions (T) is shown. **D.** There is (almost) no mismatch between actions (T) and beliefs (t), in contrast to what is observed for the ICG. There is apparently a slightly pessimistic bias at $k \in \{1,2,3\}$.

SI References

1. McKelvey, R.D., and Palfrey, T.R. (1992). An experimental study of the centipede game. Econometrica, 803-836. https://doi.org/10.2307/2951567.
2. Stewart, A.J., and Plotkin, J.B. (2013). From extortion to generosity, evolution in the iterated prisoner's dilemma. Proceedings of the National Academy of Sciences *110*, 15348-15353. https://doi.org/10.1073/pnas.1306246110.
3. Stewart, A.J., and Plotkin, J.B. (2014). Collapse of cooperation in evolving games. Proceedings of the National Academy of Sciences *111*, 17558-17563. https://doi.org/10.1073/pnas.1408618111.
4. Fey, M., McKelvey, R.D., and Palfrey, T.R. (1996). An experimental study of constant-sum centipede games. International Journal of Game Theory *25*, 269-287. https://doi.org/10.1007/BF02425258.
